# Loss of neurogenesis in *Hydra* leads to compensatory regulation of neurogenic and neurotransmission genes in epithelial cells

**DOI:** 10.1098/rstb.2015.0040

**Published:** 2016-01-05

**Authors:** Y. Wenger, W. Buzgariu, B. Galliot

**Affiliations:** Department of Genetics and Evolution, Institute of Genetics and Genomics in Geneva (IGe3), Faculty of Sciences, University of Geneva, 30 quai Ernest Ansermet, CH-1211 Geneva 4, Switzerland

**Keywords:** *Hydra* adult neurogenesis, plasticity of epithelial cells, interstitial stem cells, RNA-seq transcriptomics, flow cytometry cell sorting, hydroxyurea

## Abstract

*Hydra* continuously differentiates a sophisticated nervous system made of mechanosensory cells (nematocytes) and sensory–motor and ganglionic neurons from interstitial stem cells. However, this dynamic adult neurogenesis is dispensable for morphogenesis. Indeed animals depleted of their interstitial stem cells and interstitial progenitors lose their active behaviours but maintain their developmental fitness, and regenerate and bud when force-fed. To characterize the impact of the loss of neurogenesis in *Hydra*, we first performed transcriptomic profiling at five positions along the body axis. We found neurogenic genes predominantly expressed along the central body column, which contains stem cells and progenitors, and neurotransmission genes predominantly expressed at the extremities, where the nervous system is dense. Next, we performed transcriptomics on animals depleted of their interstitial cells by hydroxyurea, colchicine or heat-shock treatment. By crossing these results with cell-type-specific transcriptomics, we identified epithelial genes up-regulated upon loss of neurogenesis: transcription factors (*Dlx*, *Dlx1*, *DMBX1/Manacle*, *Ets1*, *Gli3*, *KLF11*, *LMX1A*, *ZNF436*, *Shox1*), epitheliopeptides (*Arminins*, *PW peptide*), neurosignalling components (*CAMK1D*, *DDCl2*, *Inx1*), ligand-ion channel receptors (*CHRNA1*, *NaC7*), *G-Protein Coupled Receptors* and *FMRFRL.* Hence epitheliomuscular cells seemingly enhance their sensing ability when neurogenesis is compromised. This unsuspected plasticity might reflect the extended multifunctionality of epithelial-like cells in early eumetazoan evolution.

## 1. Introduction

The question of the origins of neurogenesis at the base of metazoans has been debated for decades. This debate was recently reinforced by reports showing the plausible sister position of ctenophores among metazoans and their divergent nervous system [1,2], implying either an independent origin of neurogenesis in ctenophores [3] or a secondary loss of the pre-synaptic equipment in Porifera [4]. Nevertheless, it is commonly accepted that cnidarians and bilaterians share a common ancestor already equipped with a robust nervous system, able to regulate neuromuscular transmission and possibly sensory organs [5–8]. Among cnidarians, the regulation, function and dynamics of neurogenesis were deeply investigated in the freshwater hydrozoan polyp named *Hydra.* In this study we tested the plasticity of non-neuronal tissues, i.e. epitheliomuscular cells and gland cells, in response to the loss of neurogenesis in an adult organism. Although epitheliomuscular cells never spontaneously differentiate into nerve cells in *Hydra*, adult animals survive the loss of neurogenesis if they are maintained alive by force-feeding once neurons have disappeared. This study is the first attempt to characterize the molecular components that are candidates for supporting epithelial plasticity in early-branched eumetazoans.

*Hydra* is formed of two cell layers, the epidermis and the gastrodermis, which house specific epitheliomuscular cell populations, named ectodermal epithelial and endodermal epithelial, respectively. These epithelial populations, which do not mix and cannot replace each other, share important properties [9]. Epitheliomuscular cells are fully differentiated multifunctional cells, which concomitantly behave as stem cells, continuously self-renewing when located in the body column (bc). With tissue growth, epithelial cells are slowly displaced towards the extremities, where they stop cycling and terminally differentiate when reaching the head region at the apical pole, or the foot region at the basal pole [9–13]. In complement to these two epithelial populations, a sophisticated nervous system, made of sensory–motor neurons, ganglionic neurons and mechanosensory cells named nematocytes, regulates the various behaviours of *Hydra* such as contraction bursts, touch response, light response, feeding behaviour, walking and swimming [5,8,14–19]. These cells differentiate from non-epithelial multi-potent stem cells named interstitial stem cells (i-cells). Interstitial cells are located in the central half of the animal, where they continuously self-renew, producing progenitors during the whole life of the animal. As a result, the central body column of the adult *Hydra* polyp is neurogenic, whereas the extremities contain a dense and highly differentiated nervous system [20].

Remarkably, *Hydra* survives the elimination of i-cells: several weeks after a transient exposure to hydroxyurea (HU) [21] or colchicine (Col) [22], animals become ‘nerve-free’ or epithelial. Similarly, the natural thermosensitive strain *sf-1* (*Hv_Sf1*) eliminates cycling interstitial cells upon heat-shock (HS), becoming nerve-free after several weeks [23,24]. Epithelial animals no longer respond to mechanical stimulations and as a result cannot use their tentacles to catch food [21,22], but they still maintain some excitability and spontaneous pacemaker activity [25]. Interestingly such drug- or HS-induced nerve-free animals maintain their ability to regenerate after bisection, or even reproduce through budding when force-fed in the laboratory [26,27]. These puzzling observations suggested that the nervous system has a limited or no impact on developmental processes because those are carried exclusively by epithelial cells in nerve-free adult *Hydra* polyps. It is possible, however, that the behaviour of epithelial cells differs between nerve-free and homeostatic contexts, i.e. that epithelial cells adapt to the loss of i-cells by enhancing some sensing/acting functions so that the animal can remain fit, survive and develop when necessary.

To investigate the putative adaptation process of the epithelial cells at the genetic level, we performed a systematic analysis of the expression of the neurogenesis (NG) and neurotransmission (NT) genetic programmes in neurogenesis-free animals generated by three different methods: HS, HU or Col treatments all performed in the thermosensitive *Hv_Sf1* strain. In short, we performed three distinct series of transcriptomic analyses to (i) map the spatial NG and NT gene expression profiles along the body axis, (ii) measure the response of NG and NT genes to the loss of neurogenesis after either drug (HU or Col) or HS treatments and (iii) assess which cell types express the NG and NT genes. We focused on the modulations displayed by 193 genes involved or predicted to be involved in neurogenesis, such as signalling cascades, transcription factors (TFs), RNA-binding proteins, and 376 genes involved or predicted to be involved in neurotransmission, such as peptides, ligand-gated ion channels (LICs), G-protein coupled receptors (GPRs), neurotransmitter biosynthetic enzymes and synaptic proteins (see electronic supplementary material table S1). All together, the results presented here are evidence of the changes undergone by the epithelial cells to overcome the loss of i-cells, and reveal a series of candidate ‘plasticity’ genes. The observed modulations suggest that epitheliomuscular cells in *Hydra* are highly plastic, undergoing sustained modification of their transcriptional programme after the elimination of interstitial cells.

## 2. Material and methods

### (a) *Hydra* culture and drug treatment

**Figure 1.**
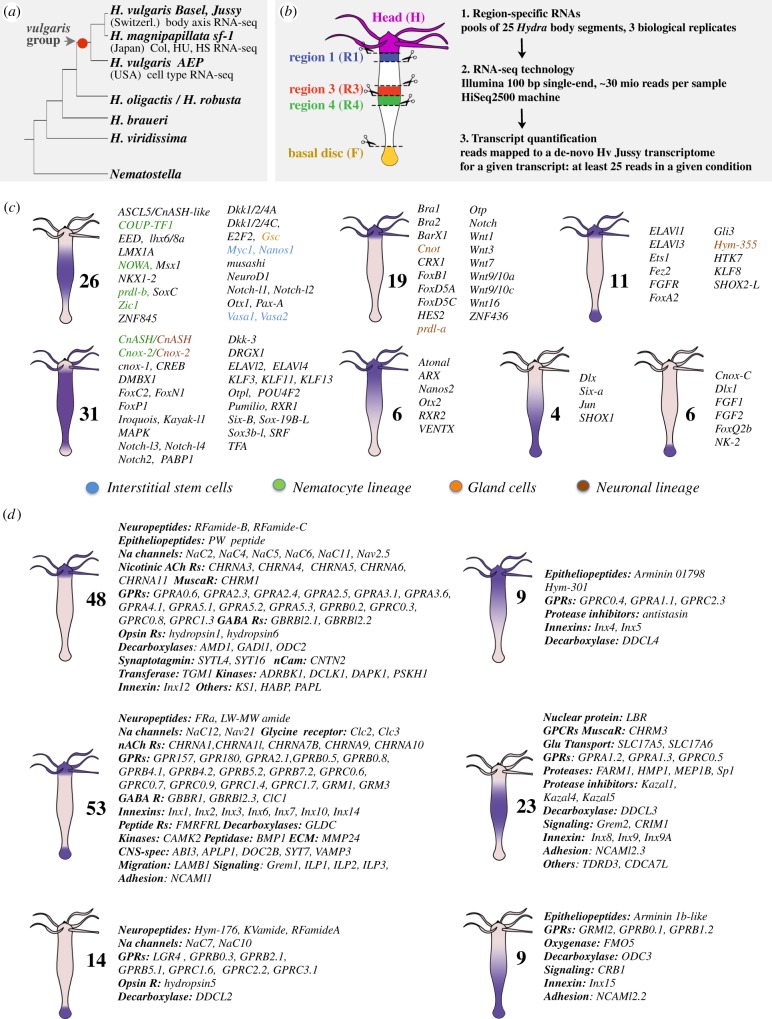
Quantitative RNA-seq analysis of the spatial expression patterns of genes predicted or identified as playing a role either in neurogenesis (NG) or in neurotransmission (NT) in *Hydra*. (*a*) Schematic evolutionary tree of the *Hydra* genus. Note that all *Hydra* strains used in this study belong to the *Hydra vulgaris* (*Hv*) species. (*b*) Dissection scheme and RNA-seq procedure applied to *Hv_Jussy*. (*c*,*d*) Schematic representation of the spatial expression patterns as deduced from the RNA-seq profiles of the NG (*c*) and NT (*d*) genes along the body axis. See quantifications in electronic supplementary material, table S1.

**Figure 2.**
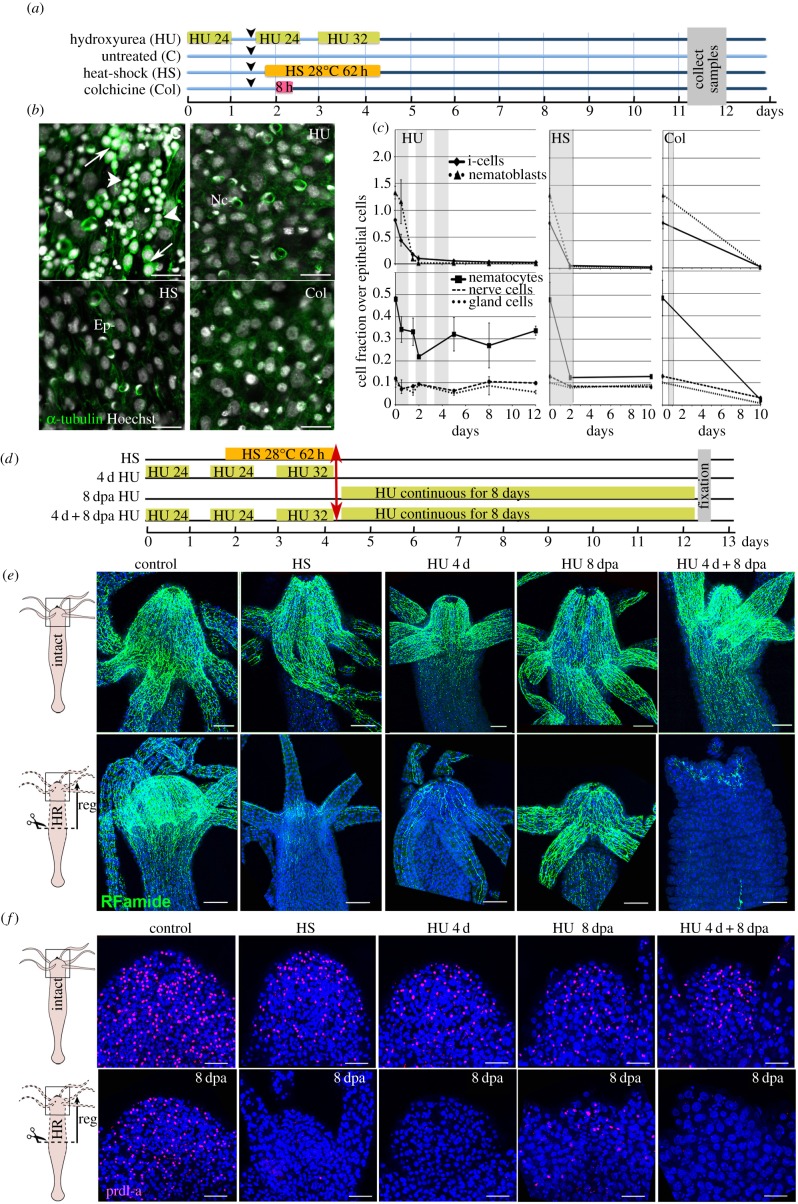
Loss of apical neurogenesis in *Hydra* after HS, HU or Col treatment. (*a*) Scheme indicating the timeline for drug treatments (HU, Col) and HS exposure of animals. Arrowheads indicate feedings. (*b*) Ectodermal view of animals fixed at day 11 after HS, HU or Col exposure as indicated in (*a*) and immunostained for *α*-tubulin (green). Note the absence of interstitial cells (arrows) and nematoblasts (arrowheads) in treated samples. Ep, epithelial cells; Nc, nematocytes, white nuclei: Hoechst staining. Scale bar: 25 µm. (*c*) Quantification of i-cells, nematoblasts (upper panels), and i-cell derivatives (lower panels) over epithelial cells in animals exposed to HS, HU or Col. Tissues were macerated and stained as in (*b*). (*d–f*) Scheme depicting the regeneration experiments conducted on HS- or HU-treated animals (*d*). Animals bisected at mid-gastric position (red arrow) were left to regenerate either in HM (conditions HS, 4 d HU), or in HM containing HU (conditions 8 dpa HU, 4 d + 8 dpa HU), then fixed at 8 dpa and immunodetected with anti-RFamide (*e*) and anti-prdl-a (*f*) antibodies. (*e*) Anatomy of the apical nervous system detected with the anti-RFamide (green) antibody in intact (upper panels) and in head-regenerating (lower panels) animals. Scale bar: 100 µm. (*f*) Neuronal progenitors and apical neurons detected with anti-prdl-a immunostaining (red) in intact (upper panels) and regenerating heads (lower panels). Scale bar: 50 µm. Note the absence of neuronal progenitors after HS, 4 d HU and 4 d + 8 dpa HU and their reduced number in the 8 dpa HU condition. Blue: DAPI staining (*e*,*f*). dpa, days post-amputation.

All animal cultures were maintained in hydra medium (HM) at 19°C, fed three times a week with freshly hatched *Artemia*, and washed 7 h after feeding and on the following day [28]. Four distinct *Hydra vulgaris* (*Hv*) strains were used in this study (figure 1*a*), two closely related strains from Switzerland, one isolated in Basel (*Hv_Basel*) and the other obtained from a single animal collected in August 2012 in Jussy, Geneva (*Hv_Jussy*, geographic coordinates: 46°15′08.8″ N, 6°16′53.5″ E). In addition, the northern American *Hv_AEP* strain was used by the Bosch laboratory (Kiel, Germany) to produce the three transgenic strains: *Ecto-GFP* (*actin::eGFP*) [29], *Endo-GFP* (*actin::eGFP*) [30] and *Cnnos1-GFP* (*Cnnos1::eGFP*) [31]. These were kindly provided to us by Thomas Bosch. Finally, the Japanese thermosensitive strain *Hv_Sf1* [24] was used for inducing the loss of neurogenesis, either with HU (Axonlab, 10 mM final) or Col (Sigma, 0.4%) treatments, or upon heat-shocking at 29°C as indicated in figure 2. Drugs were diluted in HM and animals were washed daily.

### (b) Immunodetection and whole mount mRNA *in situ* hybridization (WM-ISH)

To assess the loss of i-cells and derivatives, five to six animals were macerated as in David [32] during and after HU, Col or HS treatments in three independent experiments (figure 2*c*). After phosphate-buffered saline (PBS) washings and pre-incubation in 2% bovine serum albumin (BSA), cells were immunolabelled overnight with the anti-*α*-tubulin antibody (1 : 1000, Sigma) and detected with the anti-mouse Alexa 488 antibody (1 : 500, Life Technologies). After Hoechst nuclear staining, cells were counted using a Leica D5550 fluorescence microscope (at least 1000 per condition). The same conditions were used for *α*-tubulin whole mount immunodetection. For RFamide [33], whole animals were fixed in 4% paraformaldehyde for 4 h at 4°C, then for 2 days in methanol at −20°C. After rehydration, samples were incubated for 60 min in 0.4% Triton X-100, 2% BSA, PBS, then overnight in anti-RFamide antibody (1 : 1000, kind gift of C. Grimmelikhuijzen) and detected with anti-rabbit Alexa 488 antibody (1 : 600, Life Technologies). For prdl-a immunostaining [34], whole animals were fixed for 20 h at 4°C in 50% ethanol, 4% formaldehyde, washed in PBS, denaturated for 30 min in 2 N HCl, washed in PBS, pre-incubated in 2.5% BSA, immunostained with anti-prdl-a serum (1 : 1000) for 16 h at 4°C and detected with anti-rabbit Alexa 555 (1 : 400, Life Technologies). After DAPI nuclear counterstaining, samples were mounted in Mowiol and pictured on a Zeiss LSM700 confocal microscope. WM-ISH was performed according to Gauchat *et al*. [28]. We verified by WM-ISH that the *Hv* strains used in this study similarly express a subset of NT and NG genes (see electronic supplementary material, S2).

### (c) *De novo* assembly of an *Hv_Jussy* transcriptome used for spatial gene profiling

For spatial gene profiling, 25 animals from the *Hv_Jussy* strain were dissected for each replicate (figure 1*b*), each body slice being approximately 250 µm thick. All tissue samples were immediately placed in RNALater (Qiagen) and total RNA was extracted the same day (RNAeasy mini kit, Qiagen). All conditions were collected in biological triplicates over different weeks. Libraries were prepared with the Low Sample TruSeq total RNA preparation protocol from Illumina (San Diego, CA, USA). Pools of four or five multiplexed libraries were loaded per lane of a HiSeq2500 sequencer (Illumina) and single-end sequenced up to 100 nt. Before *de novo* assembly, sequencing adapters and trans-splice leaders [35,36] were removed using cutadapt [37], reads were corrected using SEECER [38] and cd-hit-454 [39]. Finally, digital normalization was performed using two rounds of the Trinity normalization tool. The resulting dataset was assembled using Trinity [40,41] with default options and Velvet/Oases [42,43]. The two assemblies were pooled and a procedure was used to reduce sequence redundancy within the dataset (see electronic supplementary material, S1 for extended details and command lines).

### (d) RNA-seq analyses upon loss of neurogenesis using the thermosensitive *Hv_Sf1* strain

**Figure 3.**
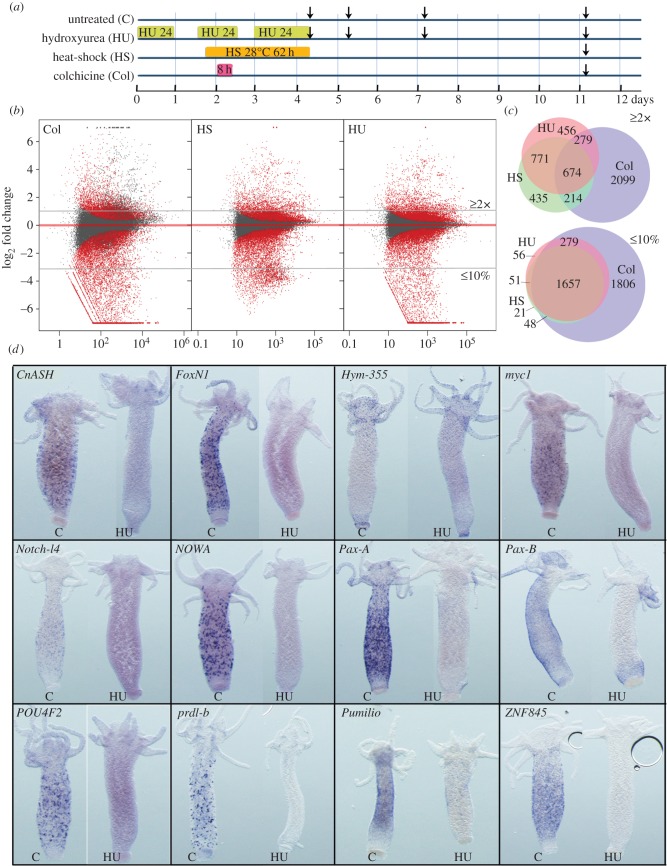
Modulations in gene expression upon loss of neurogenesis. (*a*) Timelines showing the design of the control and HU/HS/Col treatments. Vertical arrows indicate the days when body columns were dissected for RNA preparation. (*b*) MA plot of RNA-seq data 7 days after colchicine, HS or HU treatment ending. Each dot corresponds to a given transcript and red dots correspond to transcripts with significant fold change in treated versus untreated animals (*p* ≤ 0.05). *x*-axis average expression level (normalized number of reads), *y*-axis log_2_(fold change). (*c*) Venn diagrams representing the number of genes either up-regulated over 2× (upper panel), or down-regulated by more than 90% (lower panel) at day 11 after HU, HS, or Col treatments. Note that Col exposed animals up-regulate a specific subset of 2099 genes, distinct from the 674 genes found up-regulated in all three types of treatment. (*d*) Expression patterns of predicted or tested NG genes in untreated (*c*) or HU-treated (HU) intact animals as in figure 2*a*: the neuropeptide *Hym-355*, the receptor *Notch-l4*, the RNA-binding protein *Pumilio*, the TFs *CnASH*, *FoxN1*, *myc1*, *Pax-A*, *Pax-B*, *POU4F2*, *Prdl-b*, *ZNF845* and the nematocyte-specific Cys-rich *NOWA* gene. Note the complete loss of expression of all genes 7 days after HU, except *Hym-355*.

For a given condition the central body columns of 35–40 *Hv_Sf1* polyps were dissected and pooled together, from control (untreated and starved for 3, 4, 6 or 10 days), HU-treated (0, 1, 3, 7 days post-HU), HS-treated (7 days post-HS), Col-treated (10 days post-Col) animals (figure 3*a*). Each condition was sampled in three or four biological replicates, representing 37 samples in total. After trimming adapters and trans-spliced leader using cutadapt, reads from control and treated *Hv_Sf1* were mapped to the *Hv_Jussy* transcriptome (see electronic supplementary material, S1).

### (e) Stem cell-specific RNA-seq using transgenic *Hv_AEP* strains

For cell-type-specific transcriptomics, we used RNA-seq based on mRNAs from FACS-sorted cells from body columns of animals from the three *Hv_AEP* transgenic strains described above (*Ecto-GFP*, *Endo-GFP* and *Cnnos1-GFP*). Four biological replicates were prepared per condition. The body columns from 300 to 400 *Hv_AEP* transgenic polyps were dissociated with pronase (6 mg ml^−1^) in Gierer dissociation medium [44]. GFP-positive cells from the *Cnnos1-GFP* strain were sorted with a FACS Area (Beckton-Dickinson), GFP-positive cells from the *Ecto-GFP* and *Endo-GFP* strains [31,45] with a MoFlow Astrios (Beckman Coulter). The sorted cells (3 × 10^5^ to 6 × 10^5^ cells) were centrifuged, resuspended and kept in RNACell protect (Qiagen) until RNA extraction with RNeasy Plus kit (Qiagen). In addition to these FACS-sorted samples, two samples were prepared from unsorted body columns. A *de novo* transcriptome was assembled from the 12 FACS-derived samples using Trinity after adapter and trans-spliced leaders removal (cutadapt) and *in silico* reads normalization. It yielded 61 501 transcripts, arising from 44 306 putative loci (according to Trinity naming scheme). Reads trimmed with cutadapt from the different *Hv_AEP* transgenic strains were mapped to the *Hv_AEP* transcriptome for the quantification steps (see electronic supplementary material, S1).

### (f) Quantification of transcript levels and other data analysis

Mapping steps were performed separately for each library using Bowtie2 [46] with strand specificity and otherwise default options. Count tables were produced by counting the total number of mapped reads aligning to each reference sequence. Inter-sample library normalizations and statistical analyses were performed using DESeq2, v. 1.6.3 [47], with default options. Most graphs were produced using the ggplot2 [48] and ggtern packages (www.ggtern.com). When biological replicates required to be averaged (such as for ternary plots), geometric means of normalized read counts were used.

### (g) Selection of NG and NT genes expressed in *Hydra*

Beside *Hydra* genes previously characterized as neurogenic or involved in neurotransmission [5,8,14–18,49], we used the *Hydra*–human orthologome that contains 6071 sequences [50] to retrieve from the Uniprot database (www.uniprot.org/uniprot/) *Hydra* sequences orthologous to human proteins annotated either as neurogenic (signalling pathways, RNA-binding proteins, TFs), or involved in neurotransmission (synaptic machinery, GPRs, LICs, neurotransmitters, neuropeptides, metabolic enzymes, neural cell adhesion molecules). To complete this dataset we retrieved on the NCBI and Uniprot databases, and on the *Hv-Jussy de novo* transcriptome *Hydra* sequences related to sequences from any other species annotated as involved in neurogenesis or in neurotransmission. All sequences were affiliated to families using the Panther annotation system [51]. Phylogenetic analyses were performed on a number of gene families where orthology or gene naming was ambiguous. For the GPR families that are not related to bilaterian sequences, a nomenclature based on Panther affiliation and sequence clustering was established. All derived GPRs affiliated to Panther families PTHR24060, PTHR24242 and PTHR24249 are named GPRA, GPRB and GPRC, respectively. The first number in the gene name indicates the *Hydra* sub-family identified in phylogenetic trees (i.e. GPRA**1**.x), the second number indicates the protein number within the sub-family (i.e. GPRA1.**1**); the sub-family number 0 is given to sequences that take an orphan position in phylogenetic trees (i.e. GPRA0.1, GPRA0.2, GPRA0.3 are not clustered on phylogenetic trees). In total we collected and manually curated 193 NG and 376 NT sequences (available in the electronic supplementary material, table S1), some of them corresponding to isoforms of the same gene.

## 3. Results and discussion

### (a) Patterns of neurogenesis and neurotransmission along the *Hydra* body axis

*Hydra* displays a highly dynamic cellular homeostasis, characterized by an ongoing neurogenesis producing new nerve cells from interstitial progenitors. Neurogenesis is spatially regulated along the body axis, with self-renewing interstitial stem cells (i-cells) located in the central half of the animal. These cells provide committed progenitors that migrate towards the extremities where they terminally differentiate, giving rise to a dense nerve net at the apical and basal poles. By contrast, nematogenesis, i.e. the production of nematocytes from nematoblasts, which also derive from i-cells, is not spatially regulated as it takes place all along the body axis (reviewed in [5,8]). To systematically map where NG and NT genes are expressed in *Hydra*, we performed RNA-seq transcriptomics on whole tissue samples collected from five regions along the apico-basal axis of *Hv_Jussy* polyps maintained in homeostatic condition (figure 1*b*). These five regions correspond to the apical or head region (H), the upper body column (R1), the central body column (R3, R4), the peduncle and basal disc or foot (F) corresponding to the lower 20% of the animal. Thanks to the quantification of gene expression levels by RNA-seq, we obtained a reliable spatial representation of the NG and NT expression patterns.

We found a large fraction of NG genes predominantly expressed in the body column, either restricted to this region (apolar pattern) or spreading up to the extremities (ubiquitous pattern): numerous TFs such as *Cnox-2/Gsx*, *COUP-TF1*, *DMBX1*, *FoxN1*, *Gsc*, *Iroquois*, *KLF11*, *LMX1A*, *Msx1*, *NeuroD1*, *Otpl*, *Otx1*, *Pax-A*, *Pax-B*, *prdl-b*, *Six-B*, *SoxC*, *Sox3Bl*, *Zic1*, *ZNF845*, but also the RNA-binding proteins *PABP1*, *musashi*, *Pumilio*, the receptors *Notch-l1*, *Notch-l2* and the kinase *MAPK* (figure 1*c*, electronic supplementary material, S2). However, a number of TF genes exhibit a polar pattern, either strictly apical as *Cnot*, *prdl-a*, *BarX1*, *CRX1*, *ZNF436* or graded apical to basal as *Atonal*, *Nanos2*, *Otx2* or bipolar, i.e. highest at both basal and apical extremities, as *Ets1*, *KLF8* or graded basal to apical as *Dlx*, *Six-A*, *JUN*, *SHOX1,* or strictly expressed in the foot as *Dlx1*, *NK-2* (Figure 1*c*, electronic supplementary material, S2). Interestingly, the RNA-binding protein genes *Elavl1* and *Elavl3*, and the neuropeptide gene *Hym-355* are all bipolar. *Hym-355* is considered as an NG gene as its product enhances neuronal differentiation, possibly through inhibitory interactions with PW epitheliopeptides that act as neuronal inhibitors such as Hym-33H [52].

By contrast, we found a majority of NT genes strongly expressed at the poles where the nervous system is dense. As expected, the genes encoding the PW prohormone epitheliopeptide and the RFamideB and RFamideC neuropeptides, which play important roles in the feeding behaviour of the animal, are expressed at the apical pole together with *Na Channel* genes that act as receptors for RFamide peptides [18,53]. We also noted the apical expression of numerous genes encoding classes of receptors related to nicotinic acetyl choline receptors (CHRNA), metabotropic glutamate receptors (GRMs), muscarinic acetyl choline receptors (CHRMs), opsins, as well as two synaptotagmins (SYTs), several decarboxylases and the neural cell adhesion molecule 1, NCAM1 (figure 1*d*, electronic supplementary material, table S1). A second distinct large contingent of NT genes such as *neuropeptides*, *receptors* and *Innexins* exhibit a bipolar pattern, with highest expression levels observed at the apical and basal extremities, and for a more limited number of genes, restricted to the basal pole. Finally, we recorded some genes exhibiting graded patterns of expression, either from the apex, from the upper body column, or from the basal region (figure 1*d*, right panels). These graded genes encode epitheliopeptides such as arminins and Hym-301, but also proteases, protease inhibitors and innexins. In summary, this large-scale analysis, which confirms the spatial expression patterns of genes previously published, uncovers the expression of uncharacterized genes and corroborates the hypothesis stating that neurogenesis is spatially restricted to the central and paracentral regions of the body column, whereas neurotransmission is mostly active at the extremities [20].

### (b) Loss of neurogenesis differentially impacts the homeostatic and the regenerating apical nervous system

To investigate how epithelial cells adapt to the loss of neurogenesis, we used three well-established procedures that deplete the stock of cycling interstitial cells and abolish neurogenesis in *Hydra*, either chemically with HU [21] or Col [22] treatments, or physically through HS applied to the thermosensitive strain *Hv_Sf1* [24,27] (figure 2*a*)*.* HU and Col both inhibit cell cycle progression, although at distinct phases, DNA replication for HU, microtubule polymerization and mitotic progression for Col. As a consequence, cycling interstitial cells undergo cell death within 2 days, similarly to the heat-sensitive cycling interstitial cells of the *Hv_Sf1* strain upon HS [54,55]. To verify the efficiency of these procedures, we examined the cellular composition of epidermis of animals exposed to one or the other treatment and we noted the absence of i-cells and nematoblasts 7–10 days after treatment (figure 2*b*). To quantify the loss of interstitial cells, we macerated *Hydra* tissue at various time points after HS, HU or Col treatment, and in agreement with previous reports, we noted already after the first HU pulse a drastic decrease in i-cells and nematoblasts (figure 2*c*). Seven days after the third HU pulse or after HS treatment (i.e. on day 11), i-cells, which normally represent 20–26% of the total cell number [54], decrease to less than 2% and nematoblasts are completely absent, whereas nematocytes, nerve and gland cells are still present. The effect of Col treatment is more pronounced, also affecting the differentiated cells of the interstitial lineage (nematocytes, neurons, gland cells) whose number is rapidly reduced (figure 2*c*). In conclusion, all treatments efficiently deplete the stock of interstitial cycling cells.

In homeostatic condition, nerve cells are continuously produced from interstitial progenitors that are located in the upper body column and in the peduncle region (lower body column), regions from which they migrate towards the extremities where they terminally differentiate and form nerve nets. To investigate the potential spatial reorganization of nerve nets upon the loss of neurogenesis, we analysed the bipolar expression pattern of the RFamide neuropeptide, which is produced by a subset of apical and basal neurons [33]. To monitor the loss of neurogenesis in homeostatic and regenerated tissues exposed to HU or HS, we also used the anti-prdl-a antibody, which was raised against a homeoprotein expressed in neuronal progenitors and nerve cells of the apical region (figure 2*d*) [34]. In non-regenerative conditions, HS or HU treatment does not readily affect the pre-existing apical RFamide pattern, still detected in the hypostome and tentacles at day 12 (figure 2*e*, upper panels), although dendrites appear altered (not shown). Similarly, the nuclear prdl-a expression pattern appears roughly unaffected in HS-treated or HU-treated non-regenerating polyps when compared with controls (figure 2*f*, upper panels).

By contrast, the formation of the apical nerve net after mid-gastric bisection is drastically impaired when HS or HU treatments are applied before amputation (figure 2*e*,*f*, lower panels). In such animals the newly formed head contains very few RFamide**^+^** neurons and no prdl-a**^+^** nuclei, whereas untreated animals exhibit RFamide**^+^** and prdl-a**^+^** neuronal populations similar to that observed in non-amputated animals (figure 2*e*,*f*). The most dramatic phenotype is observed in animals continuously exposed to HU before and during regeneration (4 d + 8 dpa), these are unable to fully regenerate their head and show very few RFamide**^+^** cells at the apex. Surprisingly, when HU treatment is started at the time of amputation (HU 8 dpa), the RFamide pattern is comparable with that of untreated animals, even though fewer prdl-a**^+^** cells are detected in the regenerated head (figure 2*e*,*f*). This last result confirms that the proapoptotic effects of HS or HU on cycling cells are not immediate, leaving enough time for interstitial progenitors to undergo neuronal differentiation after amputation and thus survive.

### (c) Loss of neurogenesis differentially impacts the homeostatic and the regenerating basal nervous system

**Figure 4.**
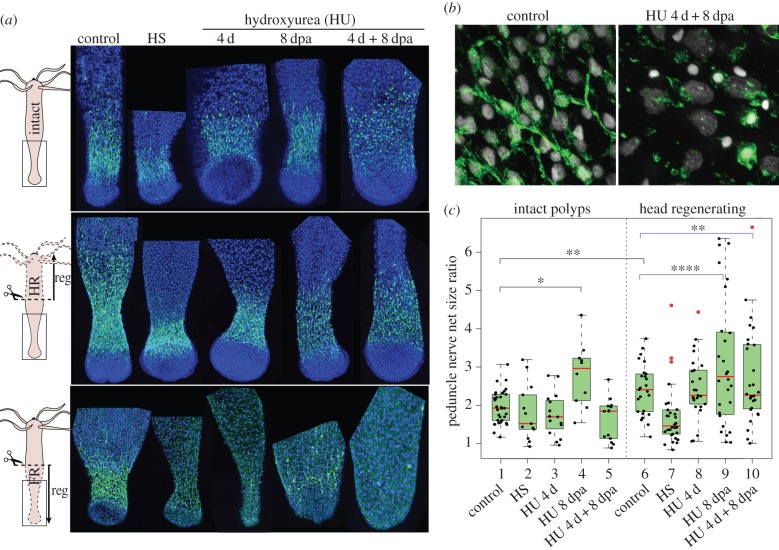
Loss of basal neurogenesis in *Hydra* after HU exposure. (*a*) Anatomy of the basal nervous system detected with anti-RFamide immunostaining (green) in intact polyps (upper), in lower halves having regenerated their apical region for 8 days (middle) or in upper halves having regenerated their basal region for 8 days (lower). Conditions of HU treatment are shown in figure 2*d*. (*b*) Higher magnification of the basal nerve net in untreated and HU-treated (4 d + 8 dpa) intact animals. (*c*) Modulations of the RFamide basal index in intact and head-regenerating polyps. Black brackets indicate the statistical testing on mean values calculated with two-sided Welch *t*-test and blue brackets indicate statistical testing on the variance between two populations calculated with the *F*-test (*p < 0.05; **p < 0.01; ****p < 0.0001).

We performed similar analyses on the second nerve-dense region in *Hydra*, i.e. the basal nervous system located along the peduncle region of the body column (figure 4). The peduncle is characterized by a dense nerve net of RFamide**^+^** ganglionic neurons, which form a sharp boundary above the basal disc (figure 4*a*, control). In intact animals exposed to HS, HU 4 d or HU 8 dpa (figure 2*d*), the spatial organization of the nerve net was not readily modified but the neuronal morphology started to be affected (figure 4*a*, upper panel). In animals continuously exposed to HU (4 d + 8 dpa), the morphology of the RFamide**^+^** neurons is dramatically modified, with a dotted RFamide pattern in the cell soma and no RFamide expression in cellular processes that appear fragmented (figure 4*b*). To quantify the impact of HS or HU exposure on the basal nerve net, we measured the RFamide basal index, established as the ratio between the length of peduncle containing RFamide**^+^** cells and the diameter of the peduncle (figure 4*c*). This measurement confirmed that HS or 4 d HU treatments do not modify the neuronal distribution, but also showed a significant increase in the mean RFamide index value when intact animals are exposed to HU for 8 days (HU 8 dpa). As the sharp boundary with the basal disk is not modified, this result indicates that RFamide**^+^** neurons extend towards the central half of the body column upon prolonged HU treatment.

Next, we tested whether apical regeneration might influence the organization of the homeostatic basal nerve net. We noticed that untreated head-regenerating animals display an extended basal RFamide area compared with that of intact polyps (figure 4*a*, control), a finding confirmed by the measurement of the RFamide basal index in intact and head-regenerating halves (figure 4*c*). This expansion of the basal nerve net after mid-gastric amputation might be explained by the rapid migration of progenitors towards the wound [56] and their subsequent neuronal differentiation. In HS-treated animals, we noted a more compact basal RFamide**^+^** net, whereas HU given for 8 dpa or 4 d + 8 dpa in head-regenerating animals led to an extension of the basal RFamide**^+^** net together with a reduction in nerve density (figure 4*a*, middle panel; electronic supplementary material, S3). A statistical analysis on the distribution of the RFamide index (Fisher test) showed highly significant differences between the untreated and the HU 8 dpa-treated head-regenerating polyps (figure 4*c*). Hence, the expansion of the pre-existing basal nerve net is only observed when HU is applied continuously on intact or on regenerating animals, and not after HS or HU 4 d, suggesting that the continuous and the three-cycle course HU treatments differently affect the behaviour of progenitors.

We also tested the *de novo* basal neurogenesis after loss of neurogenesis as observed in foot-regenerating halves of animals exposed to HS or HU. As expected, HS and HU exposures (4 d, 8 dpa, 4 d + 8 dpa) drastically affect the formation of the nerve net with few RFamide**^+^** neurons detected in the newly regenerated foot (figure 4*a*, lower panel; electronic supplementary material, S3). Beside some progenitors that might survive HS or HU treatments, it would be interesting to monitor in each context the rate of neuron conversion from RFamide**^−^** to RFamide**^+^** nerve cells as neuronal conversion is commonly observed in *Hydra* [57–59]. Indeed Koizumi *et al*. suggested that in the absence of neurogenesis, the few RFamide**^+^** neurons observed in the newly formed head or foot might arise by conversion of pre-existing neurons displaced towards the extremities [57].

*Hydra* polyps exhibit a regular spontaneous contractile activity whose regulation is complex, relying on the autonomous activity of the epitheliomuscular cells, as well as the basal and the apical nerve nets. To show the impact of the loss of neurogenesis on the spontaneous contractile activity, we compared the contractile activity of intact animals exposed to HU to those also exposed to HU but having regenerated either their apical half or their basal half. As expected from the cellular analyses, the contractile activity of intact HU-treated animals is much higher than that of HU-treated animals lacking either their basal nervous system or their apical nervous system after regeneration (electronic supplementary material, S4, movies). In summary, these parallel investigations of apical and basal neurogenesis in *Hydra* show that two distinct modes of neurogenesis can be identified in adult *Hydra* polyps, a slow ‘homeostatic’ neurogenesis that maintains the existing apical and basal nervous systems, and a fast ‘developmental’ neurogenesis observed in regenerating tissues. The homeostatic one is not immediately affected by the elimination of i-cells and progenitors, whereas the developmental one is dramatically affected by the loss of i-cells and progenitors.

### (d) Global variations of gene expression upon loss of neurogenesis

To appreciate the impact of the loss of neurogenesis at the molecular level, we used RNA-seq to measure gene expression levels on HU, HS and Col-treated *Hv_Sf1* animals (figure 3*a*) and started by counting the number of sequences regulated upon HU, HS and Col (figure 3*b*,*c*). At the last time point sampled (day 11), HU- and HS-treated animals exhibit similar massive changes in gene expression, with over 2000 genes up-regulated at least 2×, and over 4500 genes down-regulated at least 2× (FDR ≤ 0.1), while the Col treatment affects even more genes (up: 3257, down: 8751). We found 674 genes up-regulated in the three contexts (figure 3*c*). In each context, the number of down-regulated genes exceeds the number of up-regulated ones by at least twofold, indicating that a widespread loss of transcript diversity accompanies the loss of i-cells and derivatives. In total, 3910 transcripts exhibit a reduction over 90% (HU: 2043, HS: 1769, Col: 3782), with 1657 common to all three conditions.

Among the down-regulated genes, we identified i-cell-specific genes known to be involved in neurogenesis or in the maintenance of stemness in *Hydra* and/or in other species. We investigated their expression before and after HU exposure and indeed confirmed the HU-induced down-regulation of the nematocyst gene *NOWA* [60], of the proneural gene *Achaete-Scute* named *CnASH* [61], of the paired-like gene *prdl-b* [28], of the proto-oncogene *myc1* [62] and of the regulator *ZNF845* [31]. We also characterized the interstitial-specific expression of the *Notch-like* receptor *Notchl4*, the TFs *FoxN1*, *Pax-A*, *Pax-B*, *POU4F2* and the RNA-binding protein *Pumilio* (figure 3*d*). All these genes show an expression that is either dramatically reduced or undetectable when i-cells and progenitors are eliminated. *PaxB* is no longer expressed in the body column but remains expressed in the peduncle. Similarly, the neuropeptide *Hym-355*, which is expressed in a subpopulation of apical and basal neurons [52], exhibits a persistent expression as expected from mature neurons that are still present at that time (figure 3*d*). In summary, the genes analysed above display the expected cell-type regulation, indicating that HU/HS/Col transcriptomics provide a reliable tool to monitor gene modulations linked to the loss of neurogenesis.

### (e) Epithelial expression of the NG and NT genes up-regulated after loss of neurogenesis

**Figure 5.**
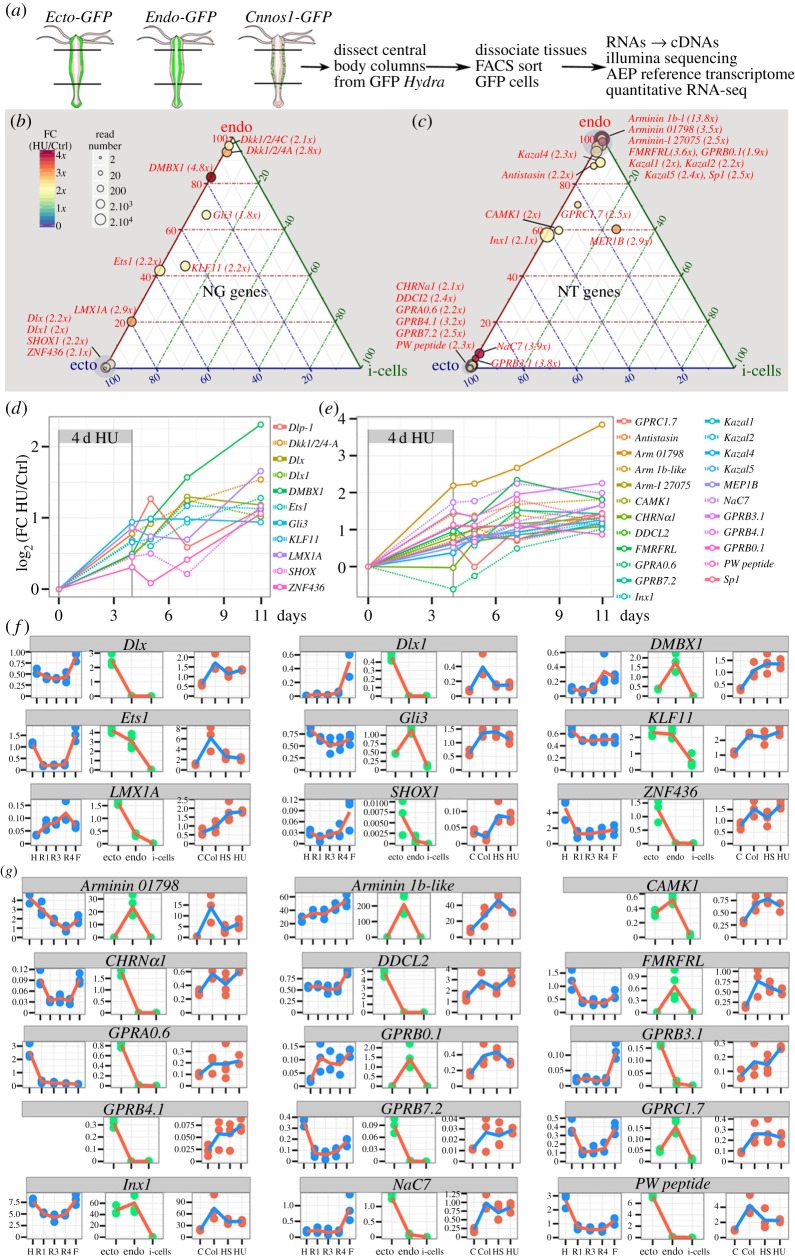
NT and NG genes up-regulated upon HU-induced loss of neurogenesis. (*a*) Scheme depicting the FACS-assisted production of cell-type-specific RNA-seq quantitative transcriptomics, ectodermal epithelial from *Ecto-GFP* [29], endodermal epithelial from *Endo-GFP* [30] and interstitial from *Cnnos1-GFP* [31]. (*b*,*c*) Ternary plots showing the results of the quantitative cross analysis of the cell-type RNA-seq datasets in homeostatic *Hv_AEP* (position within the plot) and the HU-treated RNA-seq datasets performed on *Hv_Sf1* to detect neurogenic (*b*) and neurotransmission (*c*) genes up-regulated at least twofold 7 days post HU exposure. Circle surfaces are proportional to the number of reads (see electronic supplementary material, table S1, for absolute read numbers). The fold change (FC) HU over control is given by the colour code and indicated next to gene names. (*d*,*e*) Kinetics of HU-induced up-regulation of 34 genes presumably involved in neurogenesis (*d*), through transcriptional regulation (*Dlx*, *Dlx1*, *DMBX1*, *Ets1*, *KLF11*, *LMX1A*, *ZNF436*, *Gli3*, *SHOX1*) and*/*or Wnt signalling inhibition (*Dkk1/2/4A*, *Dkk1/2/4C*), or neurotransmission (*e*), possibly neuropeptidic (*NaC7*, *FMRFRL*), epitheliopeptidic (*Arminin 1b-l*, *Arminin 01798*, *PW peptide pre-prohormone*), dopaminergic (*DOPA decarboxylase l2—DDCL*2), cholinergic (*CHRN*α*1*), or uncharacterized (*GPRA0.6*, *GPRB0.1*, *GPRB3.1*, *GPRB4.1*, *GPRB7.2*) (*e*)*.* All values at time 0 (before treatment initiation) were extrapolated to be equal to 0. (*f*,*g*) RNA-seq profiles of 9 NG and 15 NT genes up-regulated upon loss of neurogenesis. Three types of information are presented: spatial (see figure 1*b*), cell-type distribution (see *a*) and responses to Col/HS/HU treatment (see figure 2*a*). *y*-axis: thousands of mapped reads.

To map the original cell type where the NG/NT genes up-regulated after the loss of neurogenesis are expressed, we performed cell-type-specific RNA-seq transcriptomics on the epithelial ectodermal cells FACS-sorted from the *Ecto-GFP* strain [29], epithelial endodermal cells FACS-sorted from the *Endo-GFP* strain [30] and i-cells FACS-sorted from the *Cnnos1-GFP* strain [31] (figure 5*a*). To validate the results of this approach, we compared the RNA-seq predictions to the cell-type-specific expression patterns previously reported by Hwang *et al*. [63], who identified a collection of i-cell, nematoblast, nematocyte, nerve cell and gland cell-specific genes. We first found that the *Cnnos1-GFP* transcriptome appears to contain transcripts that are strictly expressed in the i-cells, and is thus devoid of contamination (electronic supplementary material, S5–S8).

By contrast, we found in the Endo-GFP transcriptome transcripts that correspond to gland cell genes (electronic supplementary material, S5C), and in the Ecto-GFP transcriptome transcripts corresponding to nematocyte, nematoblast and nerve cell genes (electronic supplementary material, S6 and S7). These results indicate some contamination of the two epithelial populations by non-epithelial cells during the dissociation/sorting process (electronic supplementary material, S6B). However, the expression level of these ‘contaminant’ genes is low when compared with their expression in the total body column as quantified using non-sorted *Hv_AEP* tissues (electronic supplementary material, S8). For example, the expression level of gland cell transcripts measured in the *Endo-GFP* fraction reaches only 8% of the expression levels measured in the whole body column. By comparison, the contamination of the Ecto-GFP transcriptome by transcripts coming from non-epithelial cells (surviving nerve cells or surviving nematocytes) is slightly more important (see electronic supplementary material, S8).

Exhibiting a typical spurious endodermal epithelial cell-type profile, the gland cell transcripts can easily be identified by the signature RNA-seq profile they show in the HU/HS/Col conditions. Gland cell genes show very low levels after Col treatment as gland cells are eliminated by colchicine, and levels are stable or elevated in HU or HS conditions (see electronic supplementary material, S9A). The 1806 transcripts that we found strongly reduced after Col but still vigorously expressed in HU/HS-treated animals (figure 3*c*) show this typical signature, indicating that they are probably expressed in gland cells. As expected, these transcripts are found in the Endo-GFP fraction, which is the only fraction containing some gland cell contaminants (electronic supplementary material, S8). All together these results indicate that modulations of the expression of epithelial and gland cell genes can be reliably traced through cell-type transcriptomics.

### (f) Candidate epithelial plasticity genes among the HU-induced upregulated NG and NT genes

To further characterize the NG and NT genes up-regulated upon the loss of neurogenesis, we focused on the genes that show a minimal twofold up-regulation after HU, HS or Col exposure in at least two of the three contexts analysed here, with high statistical support (electronic supplementary material, table S1). Following these criteria, we identified 11 genes possibly linked to neurogenesis (figure 5*b*,*d***)** and 23 to neurotransmission (figure 5*c*,*e*). Among the putative NG genes, nine are TFs, five predominantly expressed in the ectodermis (*Dlx*, *Dlx1*, *LMX1A*, *ZNF436*, *SHOX1*), two in the gastrodermis (*DMBX1*, *Gli3*) and two equally expressed in the two epithelial populations (*Ets1*, *KLF11*) (figure 5*f*). The two remaining genes encode Dickkopf-like proteins, which are produced by the gland cells and, once secreted in the gastrodermis, antagonize Wnt signalling. Their activity probably maintains neurogenesis [20]. Among the 23 up-regulated NT genes, four are restricted to the endodermal epithelial cells, encoding the two epitheliopeptides Arminin 1b-1ike and Arminin 01798, the FMRFamide-like receptor FMRFRL and the Moody-type GPR GPRB0.1; eight are strictly expressed in the ectodermal epithelial cells, encoding the epitheliopeptide PW pre-prohormone, the DOPA decarboxylase DDCL2, the receptors GPRA0.6, GPRB3.1, GPRB4.1, GPRB7.2, NaC7, CHRNA1, and three are detected in both the endodermal and ectodermal fractions, encoding the receptor GPRC1.7, the calcium/calmodulin-dependent protein kinase CAMK1D and Innexin 1 (figure 5*g*).

Two candidate NG genes and seven candidate NT genes were selected for validation by qPCR and we noted a good correlation between RNA-seq predictions and qPCR, except for the receptor GPRB4.1 that exhibited no regulation when assessed by qPCR (electronic supplementary material, S10A). Furthermore, to test whether this up-regulation is transient or sustained, we analysed the transcript levels in HU transcriptomes that were prepared at several time points after HU withdrawal (figures 3*a* and 5*d*,*f*). This analysis shows that the expression levels of the HU-induced NG and NT genes are progressively increased over time, suggesting a sustained up-regulation (figure 5*d*,*f*). In fact we found these genes still up-regulated when tested by qPCR after 23 days (not shown). Beside epithelial genes, nine up-regulated genes exhibit the typical gland cell signature, these encode the anticoagulant antistasin, the Dickkopf-related proteins Dkk1/2/4A, Dkk1/2/4C, the metalloendopeptidase MEP1B, the Ser-protease inhibitors Kazal-1, -2, -4, -5, the plasminogen-related serine protease Sp1 (see their RNA-seq profiles and ISH gland cell pattern in electronic supplementary material, S9). The gland cell-specific expression was actually independently established for *Antistasin* [64], *Dkk1/2/4A* [20], *Dkk1/2/4C* [65], *Kazal1* [66] and *Kazal2* [67].

**Figure 6.**
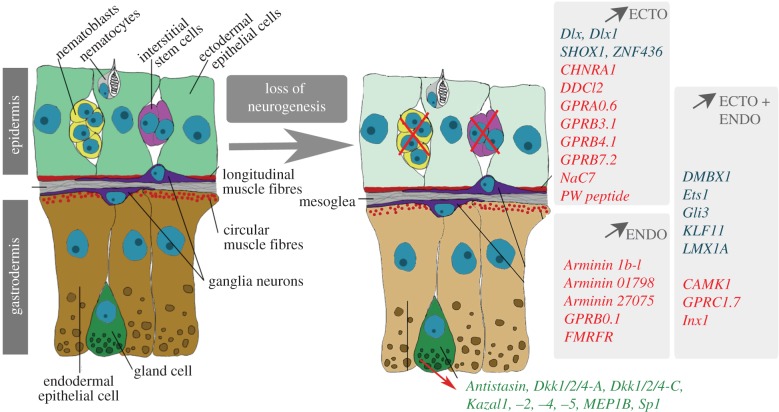
Candidate plasticity genes for epithelial adaptation to the loss of neurogenesis. Genes listed on the grey background are up-regulated at least twofold after the loss of neurogenesis, either in the ectodermal epithelial cells (ECTO, light green) or in the endodermal epithelial cells (ENDO, brown-beige) or in both (ECTO + ENDO). Genes in blue are putative neurogenic while those in red are encoding proteins likely to be involved in neurotransmission. Note the genes also up-regulated twofold in the gland cells of the gastrodermis (green). Scheme courtesy of Szymon Tomczyk.

In summary, we identified 25 epithelial and 9 gland cell genes that might support the adaptations that take place in epithelial cells when neurogenesis is abolished in *Hydra* (figure 6). Intriguingly, one finds among the most regulated genes the epitheliopeptides *Arminin 01798* and *Arminin1b-like* genes, which exhibit a strong antimicrobial activity and regulate the species-specificity of microbiomes [68,69]. This result suggests that in the absence of neurogenesis, the animal needs to adapt its defensive antimicrobial activity, or its microbiome. Alternatively, arminins might play new functions, distinct from the previous antimicrobial activities characterized so far, possibly linked to the sensing of the environment that would be necessary for the survival of ‘epithelial’ animals. Among the putative NG genes, the paired-like homeobox gene *DMBX1* exhibits the highest up-regulation. DMBX1 (diencephalon/mesencephalon homeoBox protein 1) plays an essential role for the reprogramming of mouse fibroblasts, as recently uncovered in an unbiased RNAi screen [70]. In *Hydra* where it was initially named ‘manacle’, *DMBX1* is expressed in the epithelial cells at the margin of the basal disc and asymmetrically in the budding zone [71]. As animals exposed to HU/HS/Col are starving, they do not bud and the *DMBX1* up-regulation detected in the endodermal epithelial cells of the central body column cannot be linked to budding or to basal disk formation. Therefore, we suspect that DMBX1/manacle may play unsuspected roles linked to the adaptation of epithelial cells to the loss of neurogenesis in *Hydra*.

## 4. Conclusion and perspectives

This study shows that the loss of neurogenesis in *Hydra* directly impacts the surrounding epithelial cells by modifying the genetic programmes they express. By crossing the information obtained in three types of transcriptomic approaches, spatial along the body column, cellular on three distinct FACS-sorted cell populations, and pharmacological in animals exposed to drugs or heat-shock, we identified 25 genes expressed in epithelial cells of the neurogenic body column in homeostatic conditions, which exhibit a minimal twofold up-regulation in the contexts where neurogenesis was inactivated (figure 6). This up-regulation is steadily increasing over 11 days following HU treatment initiation and still elevated after 23 days (not shown), suggesting a permanent adaptation. Interestingly, a similar epithelial up-regulation was previously described for the *rack1* gene, which encodes a WD-type protein kinase C receptor, in nerve-free animals 3 weeks after HU treatment [72]. In untreated animals *HvRack1* is expressed predominantly in gland cells and interstitial cells, and to a lesser extent in digestive cells, indicating that compensatory expression of interstitial-specific genes in epithelial cells is possible upon loss of the interstitial lineage. In this study, we also identified nine genes expressed in gland cells which are up-regulated after the elimination of i-cells (figure 6). As gland cells survive for weeks after the loss of i-cells, this result suggests that gland cells play a role in the epithelial plasticity.

Several important questions need to be addressed following the observations reported here. Firstly, the relevance of the observed gene up-regulations to the adaptation of *Hydra* to the loss of neurogenesis needs to be confirmed by functional studies. If these gene modulations are important for animal survival, we predict that silencing one or several of these 34 genes will either affect the survival of the animals and/or the maintenance of their developmental programmes. Secondly, we have investigated here the impact of the systemic loss of neurogenesis on epithelial cells but we expect similar modifications when i-cells are locally eliminated as observed in head-regenerating tips after mid-gastric bisection [56]. If confirmed, the local injury-induced epithelial plasticity might also participate in head regeneration. Thirdly, the cellular functions linked to the elevated levels of these NG/NT genes need to be explored. Epithelial cells play multiple functions in *Hydra* [9] and several of them might be affected by these genetic changes, such as cell-to-cell communication, epithelial conduction through gap junctions, cell adhesion, cell cycle regulation or differentiation. As an example, *LMX1A*, which regulates neuronal differentiation in mammals, might potentially enhance neuronal-like functions of epithelial cells, such as an increase of their sensitivity and response to environmental signals. Fourthly, as upon elimination of the cycling interstitial cells a number of genes are found up-regulated in a few days, we suspect that, in control animals, these genes are maintained repressed by signals from the surrounding i-cells and/or the interstitial progenitors. Such continuous crosstalk between the interstitial and the epithelial cell lineages was identified by Sugiyama and Waneck [73] who showed that elimination of the interstitial cells enhance regeneration in a regeneration-deficient mutant strain. Further studies will test this mechanism and potentially identify novel components of the signalling between interstitial and epithelial cell lineages.

Finally, deciphering the interstitial–epithelial crosstalk in *Hydra* might highlight some aspects of the origin(s) of nervous systems. Indeed, the ablation of the i-cell lineage, assumed to have a more recent origin than the epithelial one, might provide a window to reveal some facets of the proto-neuronal state of the epithelial cells. This experimental framework appears to provide a gain-of-function assay where the loss of i-cells leads to the derepression of some atavic neuronal-like functions of epithelial cells. This ‘adapted’ status might be informative to infer the ancestral status of epithelial cells in basal metazoans, i.e. a period when their multi-functionality most likely included proto-neuronal functions. The idea that ancestral multifunctional cells in basal metazoans progressively diversified into more specialized cells during evolution is largely accepted [74]. The scenario proposed by George Mackie in 1970 on the origins of neuroid conduction [75] is specially well appreciated when considering the origins of the nervous system. We propose that the loss of neurogenesis in *Hydra* provides a paradigm to test the potential of a reverse process whereby epithelial cells adapt by using an otherwise repressed ancestral toolkit to cope with the disappearance of the i-cell lineage.

## Supplementary Material

Supplementary Table 1

## Supplementary Material

Supplementary material S1-S10

## Supplementary Material

Supplementary movies

## Data accessibility

The sequence datasets supporting this article have been uploaded as part of the electronic supplementary material, table S1.
