# Supplementary material for: Loss of neurogenesis in Hydra leads to compensatory regulation of neurogenic and neurotransmission genes in epithelial cells
Source: Philos Trans R Soc Lond B Biol Sci. 2016 Jan 5;371(1685):20150040. doi: 10.1098/rstb.2015.0040 (PMC4685579; doi:10.1098/rstb.2015.0040)
Supplement: Supplementary material S1-S10 [file rstb20150040supp2.pdf]

Loss of neurogenesis in *Hydra* leads to compensatory regulation of neurogenic and neurotransmission genes in epithelial cells

Wenger Y, Buzgariu W, Galliot B

Department of Genetics and Evolution, Institute of Genetics and Genomics in Geneva (IGe3), Faculty of Sciences, University of Geneva, 30 quai Ernest Ansermet, CH-1211 Geneva 4, Switzerland.

Published in *Phil. Trans. R. Soc. B* **doi: 10.1098/rstb.2015.0040**

SUPPLEMENTAL DATA

Supplementary Table 1 (Separate file): Accession codes, Panther affiliation (PTHR), nucleotidic and protein sequences, RNA-seq expression levels of 194 neurogenesis (NG) and 374 neurotransmission (NT) genes investigated in this study

Supplemental movies (Separate file): Impact of the loss of *de novo* neurogenesis on Hydra whole body contractility (three movies)

Supplt-1: Detailed methods describing the RNA-seq assembly of the different transcriptomes and the quantification of the abundances of transcripts. 2

Supplt-2 : Comparative analysis of cell-type expression of NT and NG genes in three distinct *Hydra* strains (*Hv_Basel, Hv_Jussy, Hv_AEP*) 9

Supplt-3: Impact of HU treatment on the maintenance of the nervous system in homeostatic tissues and on the regeneration of apical and basal nervous systems in *Hydra* 11

Supplt-4: Modulations of spontaneous contractile activity in HU-treated polyps. 12

Supplt-5 : Global RNA-seq transcriptomic analysis of cell-type expression in *Hydra* and evaluation of the gland cell contamination in the Endo-GFP fraction. 13

Supplt-6: Evaluation of nematocyte contamination in the Ecto-GFP fraction. 14

Supplt-7: Evaluation of the contamination of the Ecto-GFP and Endo-GFP fractions by the nematoblasts and nerve cells 15

Supplt-8: Quantitative assessment of the non-epithelial (i-cell derivatives) contamination of the Ecto-GFP, Endo-GFP and Cnnos1-GFP cell fractions. 16

Supplt-9: Expression analysis of the gland cell genes found up-regulated after the loss of neurogenesis 17

Supplt-10: Quantitative analysis and cell-type expression of the candidates genes up-regulated upon loss of neurogenesis. 18

Supplt-1: Detailed methods describing the RNA-seq assembly of the different transcriptomes and the quantification of the abundances of transcripts.

An extended description of the procedures relating to RNA-seq experiments are provided in sections S1.1 to S1.5. Further details concerning the command lines and program versions are provided in section S1.6.

S1.1 De-novo assembly of the Hv_Jussy transcriptome used for spatial gene profiling and drug / heat-shock treatments

For spatial gene profiling, 25 animals from the *Hv* Jussy strain were dissected for each replicate as depicted in **Fig. 1B**, each body slice being ~250 µm thick, and reads were mapped to a *Hv_Jussy de-novo* transcriptome. All tissue samples were immediately placed in RNALater (Qiagen) and total RNAs were extracted the same day (RNAeasy mini kit, Qiagen). All conditions were collected in biological triplicates over different weeks. An average of ~30 (± 2.5 SD) million reads per sample was sequenced. Before *de-novo* assembly, sequencing adapters and trans-splice leaders (Stover and Steele 2001; Derelle, et al. 2010) were removed using cutadapt (Martin 2011), reads were corrected using SEECER (Le, et al. 2013) and cd-hit-454 (Li and Godzik 2006). Finally, digital normalization was performed using two rounds of the Trinity normalization tool. The resulting dataset was assembled using Trinity (Grabherr, et al. 2011; Haas, et al. 2013) with default options and Velvet/Oases (Zerbino and Birney 2008; Schulz, et al. 2012) using a kmer value of 35 and final coverage of 5. As post-processing, the velvet (430’345 sequences) and trinity (773’456) assemblies were pooled and, for each sequence, the longest open reading frame (ORF) of more than 100 amino acids encoded by the forward strand was extracted using EMBOSS tools (Rice, et al. 2000). Near duplicates sequences were removed using a 98% similarity threshold on both amino acids and nucleotides Cd-hit and Cd-hit-est (Li and Godzik 2006) yielding 131’430 sequences. Finally, only sequences aligned by blast (Camacho, et al. 2009) that match the *Hm-105 strain* genome (*Hydra vulgaris* group) (Chapman, et al. 2010) with at least 90% similarity and E-values lower than 10^{-10} were selected for further steps (107’422 sequences).

Discriminating between sequences originating from the same gene (e.g. alleles or natural variants) and close paralogs is a difficult task in absence of a comprehensively assembled genome. In order to facilitates this aspect in downstream analyses, remaining sequences were realigned to the *Hm-105 strain* genome (Chapman, et al. 2010) using GMAP (Wu and Watanabe 2005) and assigned to genomic loci, considering here that one single base overlap was sufficient for an assignation into the same locus. A final round of clustering was performed *per locus* with a 95% sequence identity threshold on deduced protein sequences. The assembly resulted in 79’773 sequences (51’633 and 28’140 from the Trinity and Velvet/Oases assembly, respectively), arising from 25’878 loci.

S1.2 RNA-seq analyses upon loss of neurogenesis using the thermosensitive Hv_Sf1 strain (HU/HS/Col treatments)

For a given condition the central body columns of 35-40 *Hv_Sf1* polyps were dissected and pooled together, from untreated (3, 4, 6 and 10 starvation days), HU-treated (0, 1, 3, 7 days post-HU), HS-treated (7 days post-HS), Col-treated (10 days post-Col) animals (**Fig. 4A**). Each condition was sampled in 3 or 4 biological replicates, representing 37 samples in total. Libraries were prepared with the Low Sample TruSeq total RNA preparation protocol from Illumina (San Diego, CA, US). Pools of 4 or 5 multiplexed libraries were loaded per lane of a HiSeq2500 sequencer (Illumina) and single-end sequenced up to 100 nt. After trimming adapters and trans-spliced leader using cutadapt, reads from control and treated *Hv_Sf1* were mapped to the *Hv_Jussy* transcriptome as the observed sequence similarities are nearly 100% between these two strains.

S1.3 Stem-cell specific RNA-seq using transgenic Hv_AEP strains (Ecto-GFP, Endo-GFP, Cnnos1-GFP)

For cell-type specific transcriptomics, we used reads obtained from FACS-sorted cells from body columns of transgenic animals obtained from *Hv_AEP* strains expressing actin::eGFP (strain called here *Endo-GFP*) (Anton-Erxleben, et al. 2009), or actin::eGFP (*Ecto-GFP*) (Wittlieb, et al. 2006) in endodermal and ectodermal epithelial cells respectively, or Cnnos1::GFP (*Cnnos1-GFP*) in i-cells (Hemmrich, et al. 2012). These strains were kindly provided to us by Thomas Bosch (Kiel, Germany). Four biological replicates were

prepared per condition. The body columns from 300-400 *Hv_AEP* transgenic polyps were dissociated with pronase (6 mg/ml) in Gierer dissociation medium (Gierer, et al. 1972). GFP positive cells from the *Cnnos1-GFP* strain were sorted with a FACS Area (Beckton-Dickinson), GFP positive cells from the *Ecto-GFP* and *Endo-GFP* strains (Hemmrich, et al. 2012; Buzgariu, et al. 2014) with a MoFlow Astrios (Beckman Coulter). The sorted cells (3.10^5 - 6.10^5) were centrifuged, resuspended and kept in RNACell protect (Qiagen) until RNA extraction with RNeasy Plus kit (Qiagen). In addition to these FACS-sorted samples, two samples were prepared from unsorted body columns. A *de-novo* transcriptome was assembled from the 12 FACS-derived samples using Trinity after adapter and trans-spliced leaders removal (cutadapt) and in-silico reads normalization. It yielded 61'501 transcripts, arising from 44'306 putative loci (according to trinity naming scheme). Reads trimmed with cutadapt from the different Hv_AEP transgenic strains were mapped to the *Hv_AEP* transcriptome for the quantification steps (see section S1.6).

S1.4 Quantification of transcript levels and other data analysis

Mapping steps were performed separately for each library using Bowtie2 (Langmead and Salzberg 2012) with strand specificity and otherwise default options. Count tables were produced by counting the total number of mapped reads aligning to each reference sequence. Inter-sample library normalizations and statistical analyses were performed using DESeq2 version 1.6.3 (Love, et al. 2014) with default options. Most graphs were produced using the ggplot2 (Wickham 2009) and ggtern packages (www.ggtern.com). When biological replicates required to be averaged (as on ternary plots), geometric means of normalized read counts were used.

S1.5. Bibliographical references

Anton-Erxleben F, Thomas A, Wittlieb J, Fraune S, Bosch TC. 2009. Plasticity of epithelial cell shape in response to upstream signals: a whole-organism study using transgenic Hydra. Zoology (Jena) 112:185-194.

Buzgariu W, Crescenzi M, Galliot B. 2014. Robust G2 pausing of adult stem cells in Hydra. Differentiation 87:83-99.

Camacho C, Coulouris G, Avagyan V, Ma N, Papadopoulos J, Bealer K, Madden TL. 2009. BLAST+: architecture and applications. BMC Bioinformatics 10:421.

Chapman JA, Kirkness EF, Simakov O, Hampson SE, Mitros T, Weinmaier T, Rattei T, Balasubramanian PG, Borman J, Busam D, et al. 2010. The dynamic genome of Hydra. Nature 464:592-596.

Derelle R, Momose T, Manuel M, Da Silva C, Wincker P, Houliston E. 2010. Convergent origins and rapid evolution of spliced leader trans-splicing in metazoa: insights from the ctenophora and hydrozoa. RNA 16:696-707.

Gierer A, Berking S, Bode H, David CN, Flick K, Hansmann G, Schaller H, Trenkner E. 1972. Regeneration of hydra from reaggregated cells. Nature New Biol 239:98-101.

Grabherr MG, Haas BJ, Yassour M, Levin JZ, Thompson DA, Amit I, Adiconis X, Fan L, Raychowdhury R, Zeng QD, et al. 2011. Full-length transcriptome assembly from RNA-Seq data without a reference genome. Nature Biotechnology 29:644-U130.

Haas BJ, Papanicolaou A, Yassour M, Grabherr M, Blood PD, Bowden J, Couger MB, Eccles D, Li B, Lieber M, et al. 2013. De novo transcript sequence reconstruction from RNA-seq using the Trinity platform for reference generation and analysis. Nature Protocols 8:1494-1512.

Hemmrich G, Khalturin K, Boehm AM, Puchert M, Anton-Erxleben F, Wittlieb J, Klostermeier UC, Rosenstiel P, Oberg HH, Domazet-Loso T, et al. 2012. Molecular signatures of the three stem cell lineages in hydra and the emergence of stem cell function at the base of multicellularity. Mol Biol Evol 29:3267-3280.

Langmead B, Salzberg SL. 2012. Fast gapped-read alignment with Bowtie 2. Nat Methods 9:357-359.

Le HS, Schulz MH, McCauley BM, Hinman VF, Bar-Joseph Z. 2013. Probabilistic error correction for RNA sequencing. Nucleic Acids Res 41:e109.

Li W, Godzik A. 2006. Cd-hit: a fast program for clustering and comparing large sets of protein or nucleotide sequences. Bioinformatics 22:1658-1659.

Love MI, Huber W, Anders S. 2014. Moderated estimation of fold change and dispersion for RNA-Seq data with DESeq2. bioRxiv.

Martin M. 2011. Cutadapt removes adapter sequences from high-throughput sequencing reads. EMBnet. journal 17:pp. 10-12.

Rice P, Longden I, Bleasby A. 2000. EMBOSS: the European Molecular Biology Open Software Suite. Trends Genet 16:276-277.

Schulz MH, Zerbino DR, Vingron M, Birney E. 2012. Oases: robust de novo RNA-seq assembly across the dynamic range of expression levels. Bioinformatics 28:1086-1092.

Stover NA, Steele RE. 2001. Trans-spliced leader addition to mRNAs in a cnidarian. Proc Natl Acad Sci U S A 98:5693-5698.

Wickham H. 2009. ggplot2: elegant graphics for data analysis. New York: Springer.

Wittlieb J, Khalturin K, Lohmann JU, Anton-Erxleben F, Bosch TC. 2006. Transgenic Hydra allow in vivo tracking of individual stem cells during morphogenesis. Proc Natl Acad Sci U S A 103:6208-6211.

Wu TD, Watanabe CK. 2005. GMAP: a genomic mapping and alignment program for mRNA and EST sequences. Bioinformatics 21:1859-1875.

Zerbino DR, Birney E. 2008. Velvet: Algorithms for de novo short read assembly using de Bruijn graphs. Genome Research 18:821-829.

S1.6 Detail of the command lines and the versions of the programs used

De-novo *transcriptome assembly and quantification of transcripts levels (Hv_Jussy strain, spatial gene profiling)*

Steps 1 to 3 were ran in parallel for every libraries. Libraries were then pooled for the remaining steps.

READS PRE-PROCESSING

1. Adapter and trans-spliced leader trimming (cutadapt v1.3)

Input file: lib001.fastq.gz (original sequencing reads)

Output file: lib001_cutadapt.fastq (cleaned reads)

```
cutadapt $(<tspl.conf) --match-read-wildcards -m 50 -q 10 lib001.fastq.gz > lib001_cutadapt.fastq
```

Content of tspl.conf file

```
-a GATCGGAAGAGCACACGTCTGAACTCCAGTCAC (5' illumina adapter)
-a CTTATTAACACAAATAGAACTGTTTGTTTATTGTGTTTTTTCCGT (reverse-complemented trans-spliced leader)
-a CTTATTAACACAAATAGAACTGTTTGTTAATTGTGTTTTTTCCGT (reverse-complemented trans-spliced leader)
-a CTTATTACACAGGGACTAAAAAGTTTCAGTATGTGTTTTTTTCCGT (reverse-complemented trans-spliced leader)
-a CTTATTACACAAAGACTAAAAAGTTTCAGTATGTGTTTTTTTCCGT (reverse-complemented trans-spliced leader)
-a CTTATTACACAGGGAATAAAAAGTTTCAGTATGTGTTTTTTTCCGT (reverse-complemented trans-spliced leader)
-a CTTATTACAAAGGGACTAAAAAGTTTCAGTATGTGTTTTTTTCCGT (reverse-complemented trans-spliced leader)
-a CTTATTACACAGAGACTAAAAAGTTTCAGTATGTGTTTTTTTCCGT (reverse-complemented trans-spliced leader)
-a CTTATTACACAGGGACTAAAAGGTTTCAGTATGTGTTTTTTTCCGT (reverse-complemented trans-spliced leader)
-a CTTATTACACAGGGACTAAAAAGTTTTAGTATGTGTTTTTTTCCGT (reverse-complemented trans-spliced leader)
-a CTTATTTACGCAATAAAACAAGAGTTAATAATGCGTTTTTTCCGT (reverse-complemented trans-spliced leader)
-a CTTATTAACACAAATTTAACGTTGAGTTTTGTTTGTGTTTTTTCCGT (reverse-complemented trans-spliced leader)
-a CTTATTGACACAAATACTTAAAAAAAGTTTAGATGTGTTTTTTTCCGT (reverse-complemented trans-spliced leader)
-a CTTATTGACACAAATACTTAAATGAAGTTTAGATGTGTTTTTTTCCGT (reverse-complemented trans-spliced leader)
-a CTTATTGACACAAATACTTAAAACAAGTTTAGATGTGTTTTTTTCCGT (reverse-complemented trans-spliced leader)
```

2. Error correction with SEECER version 0.1.3

Input file: lib001_cutadapt.fastq

Output file: lib001_cutadapt.fastq_corrected.fa

```
run_seecer.sh -t temporary_folder -k 25 lib001_cutadapt.fastq
```

3a. Individual library normalization (Trinity normalisation tool)

Input file: lib001_cutadapt.fastq_corrected.fa

Output file: lib001_cutadapt.fastq_corrected.fa_normalized_K25_C50_pctSD200.fa

```
./util/normalize_by_kmer_coverage.pl --seqType fa --JM 500G --single
lib001_cutadapt.fastq_corrected.fa --max_cov 50 --SS_lib_type F --output individual_normalization
```

3b. Merge of all individually normalized libraries

Intput file: lib001_cutadapt.fastq_corrected.fa_normalized_K25_C50_pctSD200.fa

Output file: all_libraries_normalized.fa

```
cat lib*_cutadapt.fastq_corrected.fa_normalized_K25_C50_pctSD200.fa > all_libraries_normalized.fa
```

4. Altogether re-normalization (Trinity normalisation tool version r20131110)

Input file: all_libraries_normalized.fa

Output file: all_libraries.fa_normalized_K25_C50_pctSD200.fa

```
./util/normalize_by_kmer_coverage.pl --seqType fa --JM 500G --single all_libraries.fa --max_cov 50 -
-SS_lib_type F --output altogether_normalization
```

The output has been renamed norm_reads.fa for clarity.

5. Generation of cluster consensi

5a. Cd-hit-454 (v4.6)

Input file: norm_reads.fa

Output files: cdhit_454_norm_reads & cdhit_454_norm_reads.clstr

```
cd-hit-454 -i norm_reads.fa -o cdhit_454_norm_reads -M 63000 -T 16
```

5b. Cd-hit clustering (v1.3)

Input file: cdhit_454_norm_reads & cdhit_454_norm_reads.clstr & norm_reads.fa

Output files: consensus.fasta

```
cdhit-cluster-consensus cdhit_454_norm_reads.clstr norm_reads.fa consensus.fasta output_aln
```

Typical cluster resolving an insertion possibly causing a frameshift

```
Consensus                    :  +  CTGGAGAGTCATTC-TTTTTTTTTTGCAGGAATGATCTAAGCTTGCTTCT  1-50
lib044-ST865:268:D2D32ACXX:7:1:  +  CTGGAGAGTCATTCtTTTTTTTTTTGCAGGAATGATCTAAGCTTGCTTCT
lib067-ST865:267:D2EKVACXX:4:1:  +  CTGGAGAGTCATTC-TTTTTTTTTTGCAGGAATGATCTAAGCTTGCTTCT
lib075-ST865:306:C2R0FACXX:1:1:  +  CTGGAGAGTCATTC-TTTTTTTTTTGCAGGAATGATCTAAGCTTGCTTCT
lib097-ST865:306:C2R0FACXX:7:2:  +  CTGGAGAtTCATTC-TTTTTTTTTTGCAGGAATGATCTAAGCTTGCTTCT
lib051-ST865:306:C2R0FACXX:8:1:  +  CTGGAGAGTCATTC-TTTTTTTTTTGCAGGAATGATCTAAGCTTGCTTCT
lib057-ST865:306:C2R0FACXX:3:1:  +  CTGGAGAGTCATTC-TTTTTTTTTTGCAGGAATGATCTAAGCTTGCTTCT
lib018-ST865:306:C2R0FACXX:2:1:  +  CTGGAGAGTCATTC-TTTTTTTTTTGCAGGAATGATCTAAGCTTGCTTCT
lib026-ST865:268:D2D32ACXX:4:1:  +  CTGGAGAGTCATTC-TTTTTTTTTTGCAGGAATGATCTAAGCTTGCTTCT
lib020-ST865:306:C2R0FACXX:4:2:  +  CTGGAGAGTCATTC-TTTTTTTTTTGCAGGAATGATCTAAGCTTGCTTCT
lib064-ST865:267:D2EKVACXX:4:1:  +  CTGGAGAGTCATTC-TTTTTTTTTTGCAGGAATGATCTAAGCTTGCTTCT
lib093-ST865:306:C2R0FACXX:6:1:  +  CTGGAGAGTCATTC-TTTTTTTTTTGCAGGAATGATCTAAGCTTGCTTCT
lib006-ST865:306:C2R0FACXX:7:1:  +  CTGGAGAGTCATTC-TTTTTTTTTTGCAGGAATGATCTAAGCTTGCTTCT

Consensus                    :  +  GAGTTTTTTTTAACGTTATACAGAGCTTTAGCTTTGATATTTTTGTCCTT  51-100
lib044-ST865:268:D2D32ACXX:7:1:  +  GAGTTTTTTTTAACGTTATACAGAGCTTTAGCTTTGATATTTTTGTCCTT
lib067-ST865:267:D2EKVACXX:4:1:  +  GAGTTTTTTTTAACGTTATACAGAGCTTTAGCTTTGATATTTTTGTCCTT
lib075-ST865:306:C2R0FACXX:1:1:  +  GAGTTTTTTTTAACGTTATACAGAGCTTTAGCTTTGATATTTTTGTCCTT
lib097-ST865:306:C2R0FACXX:7:2:  +  GAGTTTTTTTTAACGTTATACAGAGCTTTAGCTTTGATATTTTTGTCCTT
lib051-ST865:306:C2R0FACXX:8:1:  +  GAGTTTTTTTTAACGTTATACAGAGCTTTAGCTTTGATATTTTTGTCCTT
lib057-ST865:306:C2R0FACXX:3:1:  +  GAGTTTTTTTTAACGTTATACAGAGCTTTAGCTTTGATATTTTTGTCCTT
lib018-ST865:306:C2R0FACXX:2:1:  +  GAGTTTTTTTTAACGTTATACAGAGCTTTAGCTTTGATATTTTTGTCCTT
lib026-ST865:268:D2D32ACXX:4:1:  +  GAGTTTTTTTTAACGTTATACAGAGCTTTAGCTTTGATATTTTTGTC---
lib020-ST865:306:C2R0FACXX:4:2:  +  GAGTTTTTTTTAACGTTATACAGAGCTTTAGCTTTGATATTTTTGTC---
lib064-ST865:267:D2EKVACXX:4:1:  +  GAGTTTTTTTTA--------------------------------------
lib093-ST865:306:C2R0FACXX:6:1:  +  GAGTTTTTTTTA--------------------------------------
lib006-ST865:306:C2R0FACXX:7:1:  +  GAGTTTTTTTT---------------------------------------
```

TRANSCRIPTOME ASSEMBLY

6a. Assembly with Trinity (r20131110)

Input file: consensus.fasta

Output file: Trinity.fasta

```
Trinity --single consensus.fasta --SS_lib_type R --seqType fa --JM 250G --CPU 16 --output
trinity_assembly_cdhitconsensus --inchworm_cpu 16
```

6b. Assembly with Velvet and Oases (both v1.2.10)

Input file: consensus.fasta

Output file: transcripts.fasta

```
velveth k35_cdhit_consensus 35 -short -fasta consensus.fasta -strand_specific
velvetg k35_cdhit_consensus -read_trkg yes
oases k35_cdhit_consensus -cov_cutoff 5
```

Both velvet and trinity assemblies were pooled at this point and called all_sequences.fa

TRANSCRIPTOME POST-PROCESSING

7a. Conceptual translation using the EMBOSS tool getorf (EMBOSS v6.6.0)

Input file: all_sequences.fa

Output file: all_sequences.getorf

```
getorf -sequence all_sequences.fa -outseq all_sequences.getorf -minsize 300
```

All open reading frames of more than 300 nucleotides are obtained (possibly multiple ORFs per initial sequences). Getorf also annotate the header of each sequence with the coordinates of the detected ORF. If the ORF is encoded in the 3' → 5' direction, a (REVERSE SENSE) flag is added to the header.

```
>velvet_0000006_1 [525 - 860]
>velvet_0000006_2 [419 - 3511]
>velvet_0000006_3 [3603 - 3082] (REVERSE SENSE)
```

7b. Selection of longest ORFs on the forward strand only

```
./select_longest_from_getorf.pl all_sequences.getorf | grep "REVERSE" -v | retrieve_seq.sh -
all_sequences.getorf > clustprot.pep
```

Where:

`select_longest_from_getorf.pl`: selection of the longest ORF for a given sequence based on the information inserted by getorf in the header (see section 7a where velvet_0000006_2 would be kept in the minimalistic example provided there).

`retrieve_seq.sh`: indexes a fasta database fetch selected sequences using the cdbfasta and cdbyank (http://sourceforge.net/projects/cdbfasta/)

8. 98% identity clustering on deduced proteins (cd-hit 4.6)

```
cd-hit -i  clustprot.pep -c 0.98 -g 1 -M 0 -T 8 -n 5 -o nr98_prot.fa
grep ">.+?_\d+" nr98_prot.fa -Po | sed -r "s/^>//" | retrieve_seq.sh - all_sequences.fa >
clustDNA.fa
```

9. 98% identity clustering on RNA sequences (cd-hit-est 4.6)

```
cd-hit-est -i clustDNA.fa -c 0.98 -g 1 -M 0 -T 8 -n 10 -o nr98_DNA.fa
```

10. Blast 2.2.29+ alignment to Hm-105 genome using a 90% threshold to remove contaminants

The *Hydra* genome has been produced in two flavours. We use here the version referred in Chapman et al. 2010 as the "RP" assembly.

```
blastn -query nr98_DNA.fa -db hydra_JGI_genome.fa -num_threads 16 -evalue 1e-10 -perc_identity 90 -
outfmt 6 > blastalign
```

Any RNA sequence locally matching the genome with this blast alignment were retrieved

```
cut -f 1 blastalign | sort | uniq | retrieve_seq.sh - all_sequences.fa > align_gmap.fa
```

11. Realignment to Hm-105 genome (Hv group) using Gmap (version 2014-02-28)

```
gmap -d hydra -B 4 -t 16 -f 1 -D gmap_genome align_gmap.fa > gmap_output.txt
```

With the -f 1 option the output format is PSL. Details are given here:
http://www.ensembl.org/info/website/upload/psl.html

Based on the information retrieved by gmap, sequences were assigned genomic loci.

Briefly:

a. When a sequence matches multiple locations in the genome, as simple score consisting of the number of matches minus the number of mismatches is used to rank alignments (field1 – field2 in PSL format) and only the best alignment for a given transcript is kept. The name of the genomic contig is retrieved as well as genomic coordinates where the match occurs and the transcript name. The file is then sorted.

```
  Genomic_id     lowest_coordinate   highest_coordinate     transcript_id
Contig18519      00001084          00001800          velvet_00010305
Contig18519      00001666          00001966          velvet_00010306
Contig18519      00001789          00001966          velvet_00010307
```

b. Within matches to a genomic contig, overlapping transcripts are identified using the `lowest_coordinate` and `highest_coordinate` fields and assigned identical locus numbers.

12. Per locus 95% identity clustering on deduced proteins

```
cd-hit -i ./clusters/Locus00001.fa -c 0.95 -M 0 -T 1 -n 5 -o ./clusters/nr95.Locus00001.fa
```

The example above is given for sequences assigned to one genomic locus (Locus00001) but has been repeated for all loci containing more than one sequence (15’105 loci).

Finally, the original full-length RNA sequences (either from velvet or trinity assemblies produced as described in subsections 6a and 6b) were retrieved and the transcriptome obtained was named `hydra_transcriptome_jussy.fa`

READS MAPPING AND QUANTIFICATION

13. Quantification

After indexing of the transcriptome produced in the previous step, bowtie2 v2.2.1 was used using the command line, for each library in parallel using the output from cutadapt (see above).

```
bowtie2 --nofw -x hydra_transcriptome_jussy -U lib001_cutadapt.fastq -S 001.sam
```

The total number of reported reads mapping to all contigs were counted and reported in a count table.

De-novo *transcriptome assembly and quantification of transcript levels (**Hv_AEP** strain, stem-cell specific quantifications)*

READS PRE-PROCESSING

1. Adapter and trans-spliced leader trimming (cutadapt v1.7.1)

Input file: lib001_AEP.fastq.gz (original sequencing reads)

Output file: lib001_ AEP_cutadapt.fastq (cleaned reads)

```
cutadapt $(<tspl.conf) -m 50 -q 10 lib001_AEP.fastq.gz > lib001_AEP_cutadapt.fastq
```

The procedure was repeated for all libraries independently and all resulting fastq files were pooled into a file called `all_reads_cutadapt.fa`.

The file tspl.conf used here is strictly identical to the one used for *Hv_Jussy* containing known trans-spliced leader and illumina 3’ TruSeq adapter (see above)

READS NORMALIZATION AND TRANSCRIPTOME ASSEMBLY

2. Assembly with Trinity (r20140717)

```
Trinity --single all_reads_cutadapt.fa --SS_lib_type F --seqType fa --JM 500G --CPU 16 --
normalize_reads --output trinity_assembly --inchworm_cpu 16 --bflyCPU 16
```

This procedure resulted in the assembly of 61’501 contigs used in the study.

READS MAPPING AND QUANTIFICATION

3. Quantification

After indexing of the transcriptome produced in the previous step, bowtie2 v2.2.4 was used using the command line, for each library in parallel using the output from cutadapt (see above).

```
bowtie2  --nofw -x aep_transcriptome -U ./lib001_AEP_cutadapt.fastq -S lib001.sam
```

The total number of reported reads mapping to all contigs were counted and reported in a count table.

Quantification of transcript levels (Hv_Sf1 strain, drugs and heat-shock treatments)

READS PRE-PROCESSING

1. Adapter and trans-spliced leader trimming (cutadapt v1.7.1)

Input file: lib001_drugs_sf1.fastq.gz (original sequencing reads)

Output file: lib001_drugs_sf1_cutadapt.fastq (cleaned reads)

```
cutadapt $(<tspl.conf) -m 50 -q 10 lib001_drugs_sf1.fastq.gz > lib001_drugs_sf1_cutadapt.fastq
```

With the file tspl.conf being strictly identical to the one used for *Hv_Jussy* containing known trans-spliced leader and illumina 3’ TruSeq adapter (see above)

READS MAPPING AND QUANTIFICATION

2. Quantification

After indexing of the transcriptome produced in the previous step, bowtie2 v2.2.4 was used using the command line, for each library in parallel using the output from cutadapt (see above). The reads from *Hv_Sf1* animals were mapped to the reference *Hv_Jussy* transcriptome as described above.

```
bowtie2 --nofw -x hydra_transcriptome_jussy -U lib001_drugs_sf1_cutadapt.fastq -S lib001.sam
```

The total number of reported reads mapping to all contigs were counted and reported in a count table.

Supplt-2 : Comparative analysis of cell-type expression of NT and NG genes in three distinct *Hydra* strains (*Hv_Basel, Hv_Jussy, Hv_AEP*)

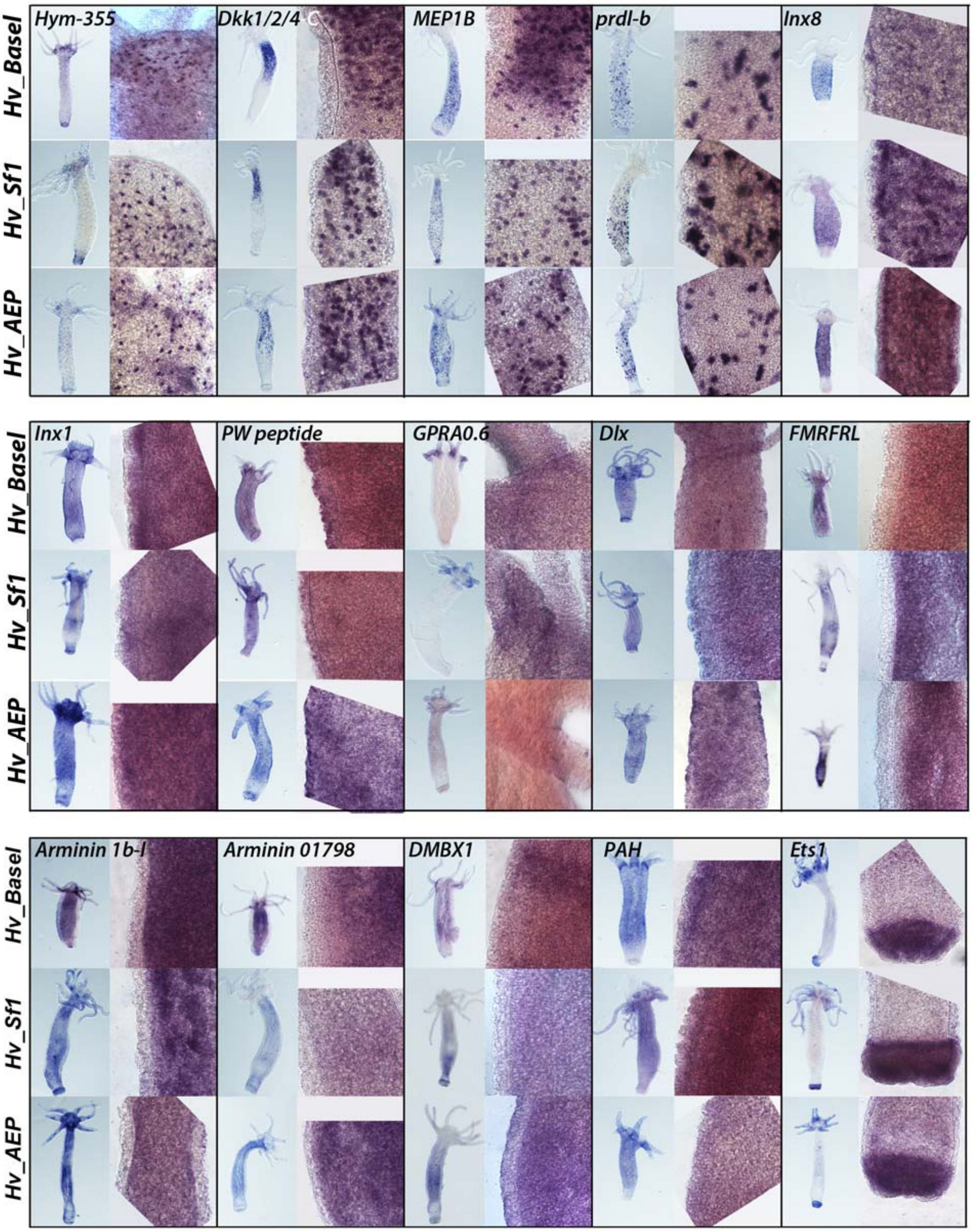

Supplt-2: Expression patterns of six NG (*Dlp-1, Dlx, DMBX1, Ets1, Hym-355, prdl-b*) and nine NT (*Arminin 1b-l, Arminin 01798, FMRFRL, GPRA0.6, Inx1, Inx8, MEP1B, PAH, PW peptide*) genes analysed by ISH in three *H. vulgaris* strains, starved for either three days (*Hv_Sf1*) or 10 days (*Hv_Basel, Hv_AEP)*. See the corresponding RNA-seq profiles in Fig. 5F, 5G and in Supplemental Table-1. All tested genes show a similar spatial expression pattern and an identical cell-type signature in the three *H.vulgaris* strains: the neuropeptide *Hym-355* in the apical and basal nerve nets, the Dickkopf related gene *Dlp-1* gradually in gland cells along the body column, the metallloendopeptidase *MEBP1* in the gland cells homogenously along body

column, the homeobox gene *prdl-b* in dividing nematoblasts, the innexin *Inx8* in the i-cells. Concerning the ectodermal epithelial cells, *Inx1*, *PW peptide* and *Dlx* are homogenously expressed along the axis ,while *GPRA0.6* transcripts are detected at the base of tentacles. The endodermal epithelial cells express the genes encoding the receptor *FMRFRl*, the antimicrobial peptides *Arminin 1b-l* and *Arminin 01798*. In contrast, the genes encoding the transcription factors *Ets1*, *DMBX1* and the *Phenyl Alanine Hydroxylase – PAH* are expressed in the epithelial cells from both the ectodermis and the gastrodemis. Note the bipolar expression pattern of *Ets1,* identified only at the base of tentacles and in the foot region of all three strains. Picturing were performed with an SZX10 Olympus steromicroscope (low magnification) and a Zeiss Axioplan2 microscope (high magnification).

Supplt-3: Impact of HU treatment on the maintenance of the nervous system in homeostatic tissues and on the regeneration of apical and basal nervous systems in *Hydra*

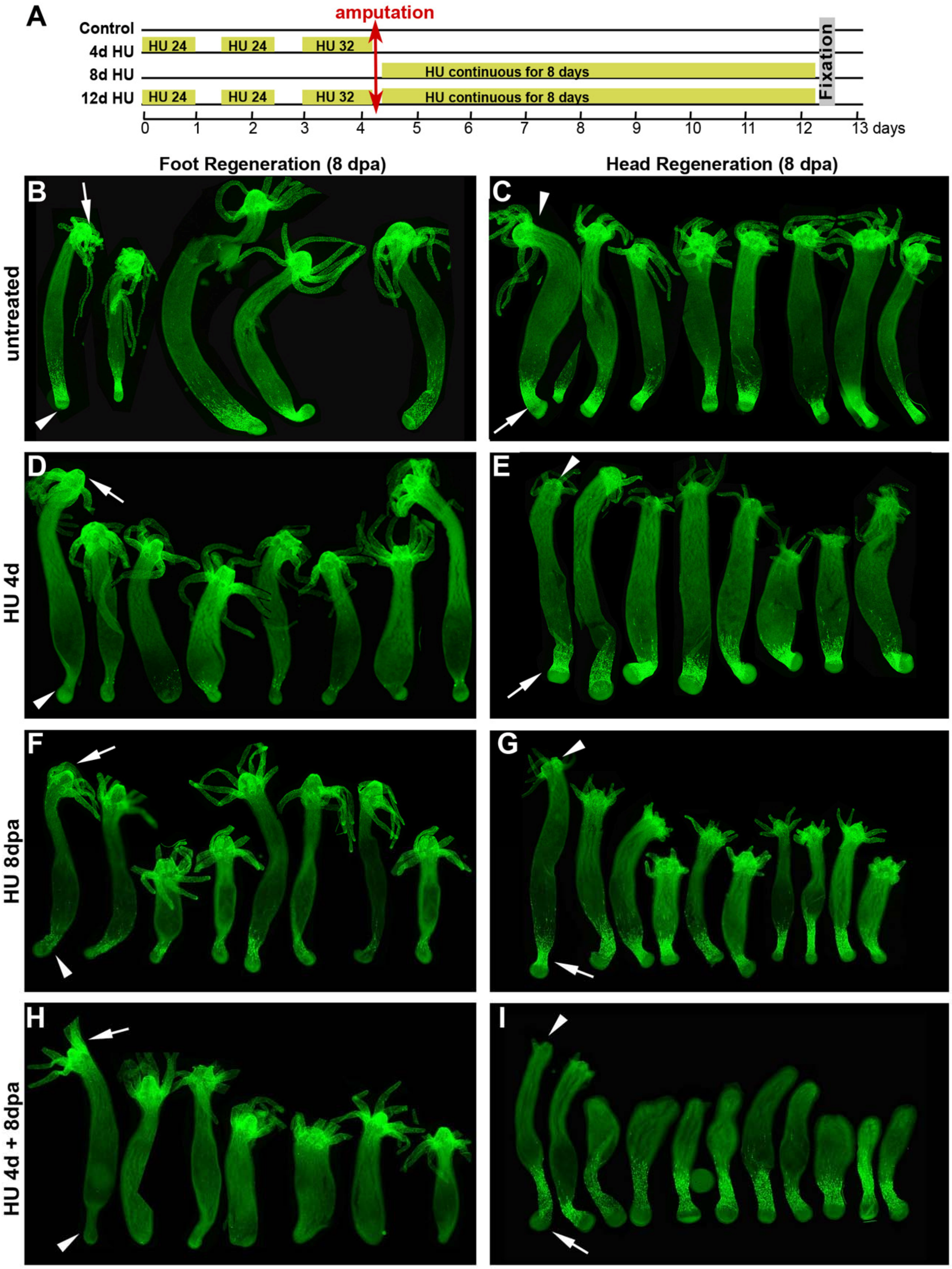

Supplt-3: In each animal, the nervous system can be observed at one pole in homeostatic (non-regenerating) condition (arrows), and at the other pole in regenerating context (arrowheads). Note that HU given before mid-gastric bisection (HU 4d) dramatically affects neurogenesis in regenerating tissues either basal (D) or apical (E) without affecting the nervous system in homeostatic tissues (apical in D and B, basal in E and C). By contrast HU given immediately after bisection (HU 8d) only slightly affects neurogenesis in regenerating tissues, either basal (F) or apical (G). Finally HU given continuously (HU 12d) affects both neurogenesis and patterning processes in regenerating tissues (H, I). Also note the extended basal nervous system in I. dpa: days post-amputation.

Supplt-4: Modulations of spontaneous contractile activity in HU-treated polyps.

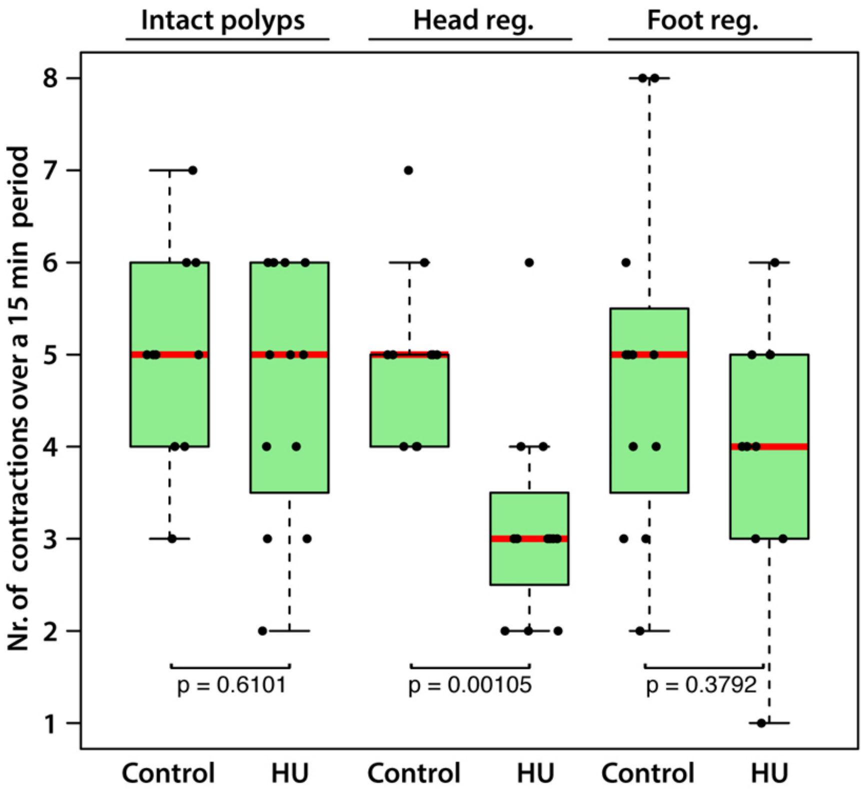

Supplt-4A: Control polyps and 4d HU-treated polyps (as indicated in Fig. 2A) were bisected at midgastric position and let to regenerate for nine days in Hydra Medium. The contractile activity of intact, head- and foot-regenerated animals was then recorded as follows: 10 animals were transferred in 1 ml HM, left for 5 minutes and recorded every 0.5 second for 15 minutes with an SZX10 Olympus stereomicroscope equipped with a DP73 Olympus camera. The number of spontaneous contraction bursts, which comprise several coordinated contractions that reduce the animals to a small ball, was counted and the contractile activity was expressed as number of contraction burst per hydra per 15 minutes. Untreated animals that regenerated their head or foot exhibit a contractile activity similar to that observed in intact polyps. As expected the HU-treated animals that regenerate heads or feet lacking a nervous system contract significantly less frequently than control animals having regenerated the same body part. However their contractile activity still persists, significantly more reduced when the apical nervous system did not regenerate properly when compared to the impact of a limited basal nervous system regeneration. A Mann–Whitney U statistical test was used to establish the p-values. See the corresponding movies as indicated below.

Supplt-4B : Three supplemental movies : Impact of the loss of *de novo* neurogenesis on *Hydra* whole body contractility

MOVIE 1: Intact polyps, either left untreated (Control) or 9 days after HU exposure (HU)

MOVIE 2: Head-regenerating polyps at 9 dpa either untreated (HR control) or 9 days post-HU (HR-HU)

MOVIE 3: Foot-regenerating polyps at 9 dpa either untreated (FR control) or 9 days post-HU (FR-HU)

Supplt-5 : Global RNA-seq transcriptomic analysis of cell-type expression in *Hydra* and evaluation of the gland cell contamination in the Endo-GFP fraction.

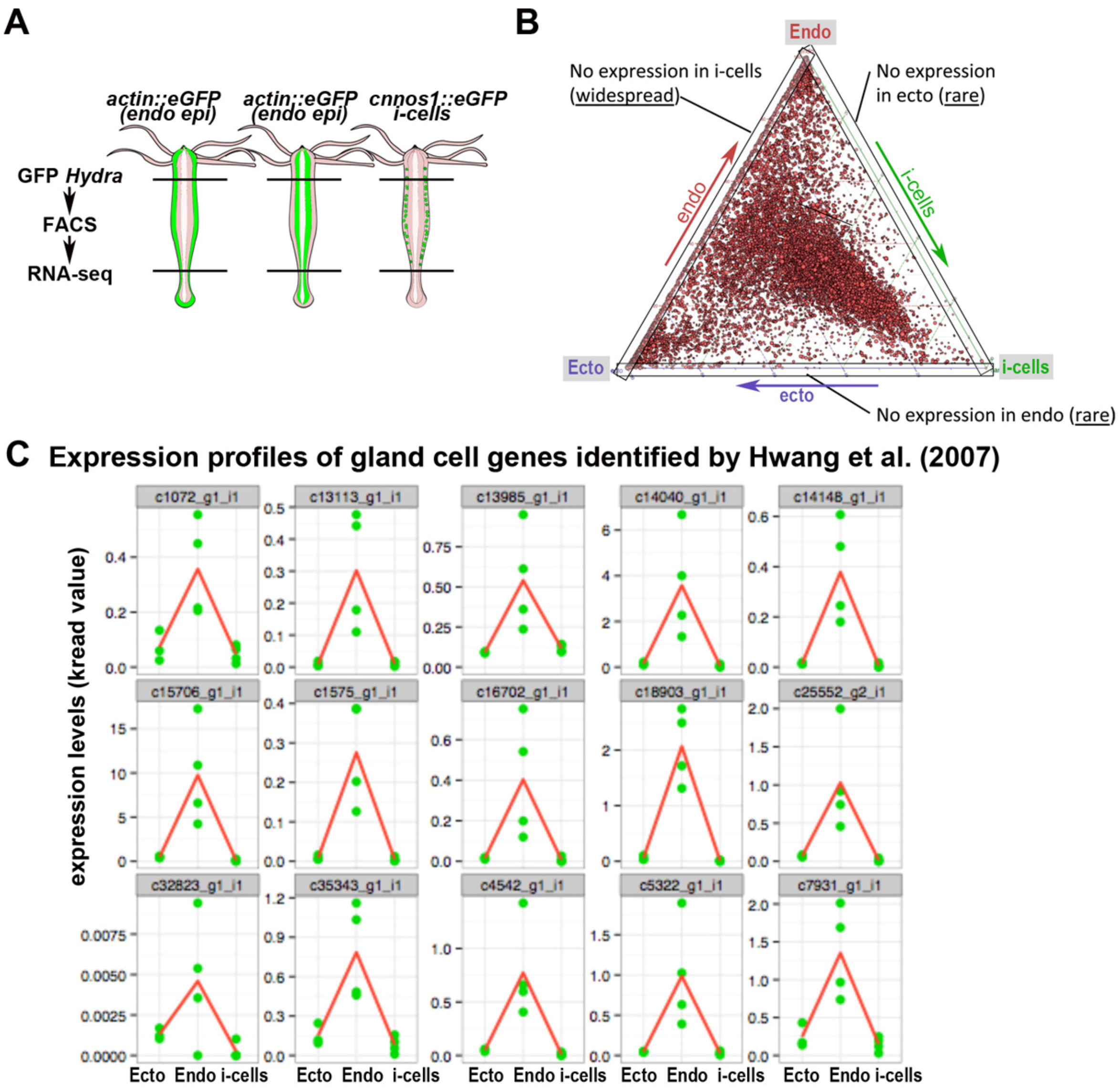

Supplt-5: (A) Scheme depicting the anatomy of the *Hv_AEP* transgenic strains used to FACS sort the GFP expressing cells, either ectodermal epithelial cells from the *Ecto-GFP* strain (*actin::eGFP*) produced by (Anton-Erxleben et al. 2009), or endodermal epithelial cells from the *Endo-GFP* strain (*actin::eGFP*) produced by (Wittlieb et al. 2006), or interstitial stem cells from the *Cnnos1-GFP* strain (*cnnos1::eGFP)* produced by (Hemmrich et al. 2012). 200-400 body columns of each strain were used for RNA extraction and Illumina sequencing (see Supplt-1). **(B)** Ternary plot representation of gene expression levels. Transcripts of a given gene (represented by a dot) distribute between the Endo-GFP (top), Ecto-GFP (left) and Ccnos1-GFP (right) transcriptomes. **(C)** Evaluation in each GFP-sorted cell population of the expression levels of gland cell genes previously characterized by Hwang et al (2007). Note that gland cell transcripts can be detected in the Endo-GFP fraction but not in the Ecto-GFP or the Cnnos1-GFP fractions. In the Endo-GFP fraction the level of expression of gland cell genes is low, below 10%, when compared to the expression level of the same genes in the total body column (see Supplt-8). Corresponding gene sequences are available upon request.

Supplt-6: Evaluation of nematocyte contamination in the Ecto-GFP fraction.

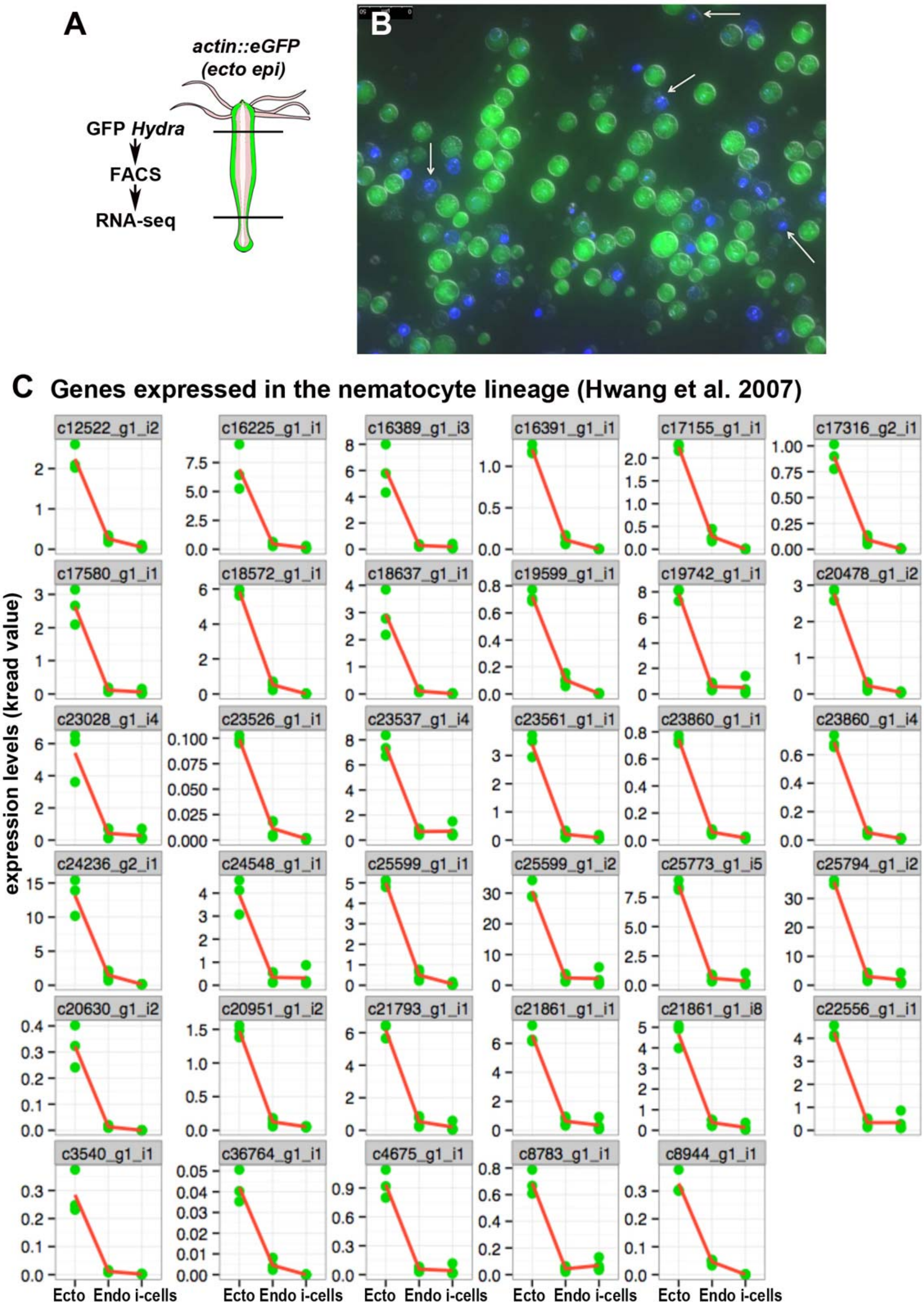

Supplt-6: **(A)** FACS sorting procedure applied to the *ECTO-GFP* strain (Anton-Erxleben et al. 2009). **(B)** View of the enrichment in GFP expressing cells immediately after flow cytometry. Arrows point to few non-GFP cells. **(C)** Expression levels of 35 genes specifically expressed in mature nematocytes as identified by Hwang et al (2007) in the Ecto-GFP, Endo-GFP and Cnnos1-GFP transcriptomes. Note the exclusive expression of these genes in the Ecto-GFP transcriptome indicating some contamination quantified in Supplt-8. Corresponding gene sequences are available upon request.

Supplt-7: Evaluation of the contamination of the Ecto-GFP and Endo-GFP fractions by the nematoblasts and nerve cells

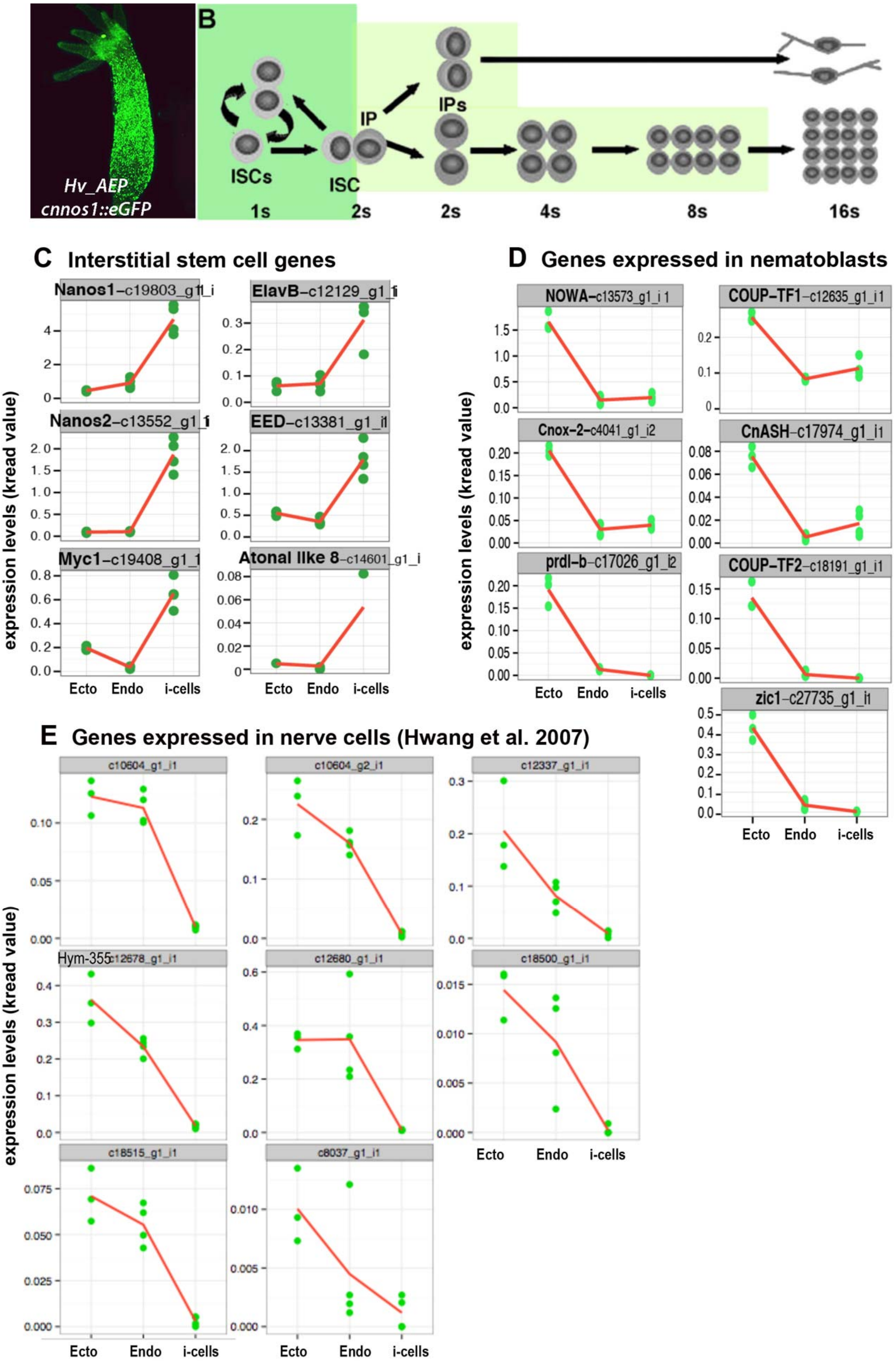

Supplt-7: (A) GFP fluorescence of a *Cnnos1-GFP_Hydra*. Note the punctuated GFP pattern restricted to the body column, typical of interstitial stem cells (i-cells). **(B)** Schematic view of the neurogenesis and nematogenesis pathways: interstitial stem cells (ISCs), interstitial progenitors (IP), nerve cells (nv, upper row), nematoblasts (nb) and nematocytes (nc, lower row). Picture and scheme are reproduced with permission from Buzgariu et al. 2014. **(C-E)** Cell-type RNA-seq profiles of i-cell (C), nb (D) and nv (E) genes. Note the absence of nb, nc and nv contamination in the Cnnos1-GFP fraction. For the quantification of the contamination of ny transcripts in the Ecto-GFP and Endo-GFP transcriptomes, see Supplt-8. Corresponding gene sequences are available upon request.

Supplt-8: Quantitative assessment of the non-epithelial (i-cell derivatives) contamination of the Ecto-GFP, Endo-GFP and Cnnos1-GFP cell fractions.

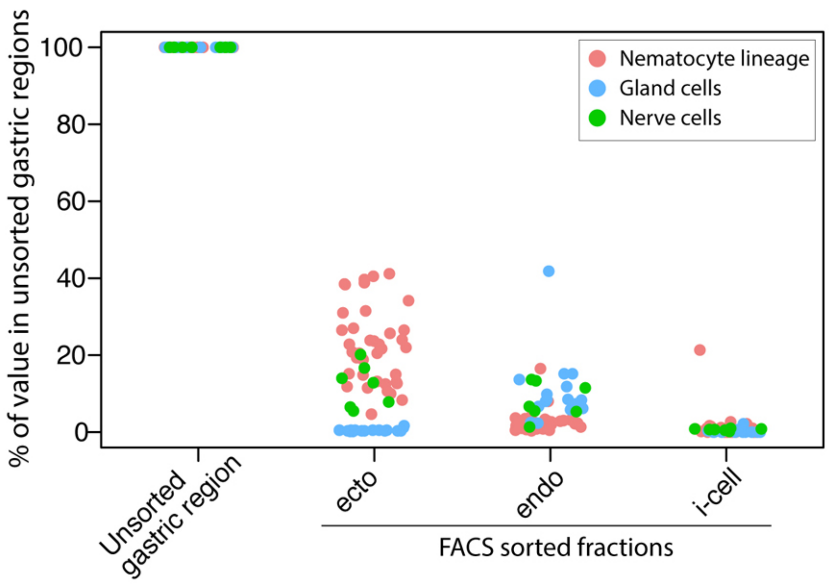

Supplt-8: Assessment of the contamination of the Ecto-GFP, Endo-GFP and Cnnos1-GFP transcriptomes by transcripts specifically detected in interstitial cell derivatives: nematocytes (coral), gland cells (blue) and nerve cells (green). Each dot corresponds to a single gene, tested in each fraction. Hwang et al. 2007 previously performed ISH on whole polyps and characterized genes as specifically expressed either in the nematocyte lineage (48, coral color), or in the gland cells (18 transcripts, blue color) or in nerve cells (8 transcripts, green color). We selected these genes and tested their RNA-seq expression levels in each cell-type specific transcriptomes (Ecto-GFP, Endo-GFP, Cnnos1-GFP) as well as. in a RNA-seq transcriptome established on whole unsorted gastric columns. The results were normalized for each gene as such as a relative expression level of 100% is obtained in the unsorted gastric column samples. The analysis shows that (i) transcripts attributed to nematocyte genes are mainly found in the Ecto-GFP fraction, (ii) transcripts attributed to gland cell genes are found in the Endo-GFP fraction, and (iii) transcripts attributed to nerve cell genes are found in both the Ecto-GFP and the Endo-GFP fractions. All these results are consistent with the location of these different cell types in the epidermis and the gastrodermis. Except for the nematocyte transcripts, these contaminations do not exceed 20% of the expression level measured in the whole body column. Finally the Cnnos1-GFP fraction appears as not contaminated.

Supplt-9: Expression analysis of the gland cell genes found up-regulated after the loss of neurogenesis

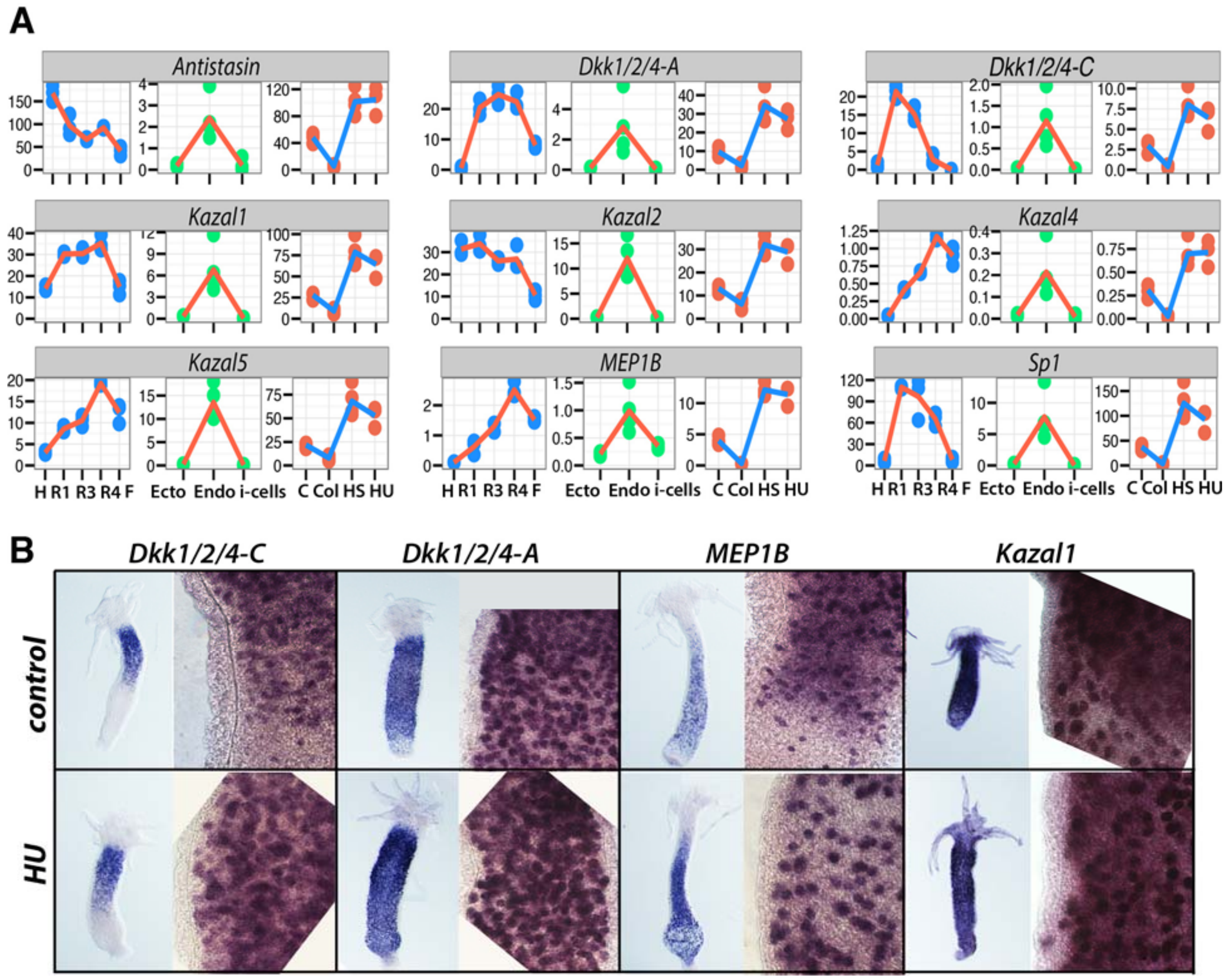

Supplt-9: (A) RNA-seq profiles of the gland cell genes identified as upregulated upon the loss of neurogenesis in drug- (HU, colchicine) or HS-treated animals (see Fig.5B-5E). The graphs show for each gene the highly variable spatial distribution of transcript levels (blue dots), the almost exclusive detection of transcripts in the Endo-GFP fraction (green dots) and the increased expression levels 11 days after HU or HS treatments but the dramatically low level of transcripts after colchicine (red dots). Y axis indicates the number of k-reads. **(B)** WM-ISH expression pattern of four gland cell genes (*Dkk1/2/4-A, Dkk1/2/4-C or Dlp1-, MEP1B, Kazal1*) in *Hv_Basel* polyps either untreated and starved for 10 days (control) or exposed to HU (as in Fig. 4A) and fixed 7 days later (HU). As previously reported by Augustin et al. (2006), *Dkk1/2/4-C exhibits* a graded expression in the gastrodermis, whereas *Dkk1/2/4-A, MEP1B* and *Kazal1* display a homogenous expression all along the body axis except the head and the foot regions. Note that upon the loss of neurogenesis, these genes are still expressed by the gland cells, which are not eliminated by the HU treatment. Quantitative assessments cannot be made from WMISH patterns as the permeability of the tissue layers decreases after HU exposure.

Supplt-10: Quantitative analysis and cell-type expression of the candidates genes up-regulated upon loss of neurogenesis.

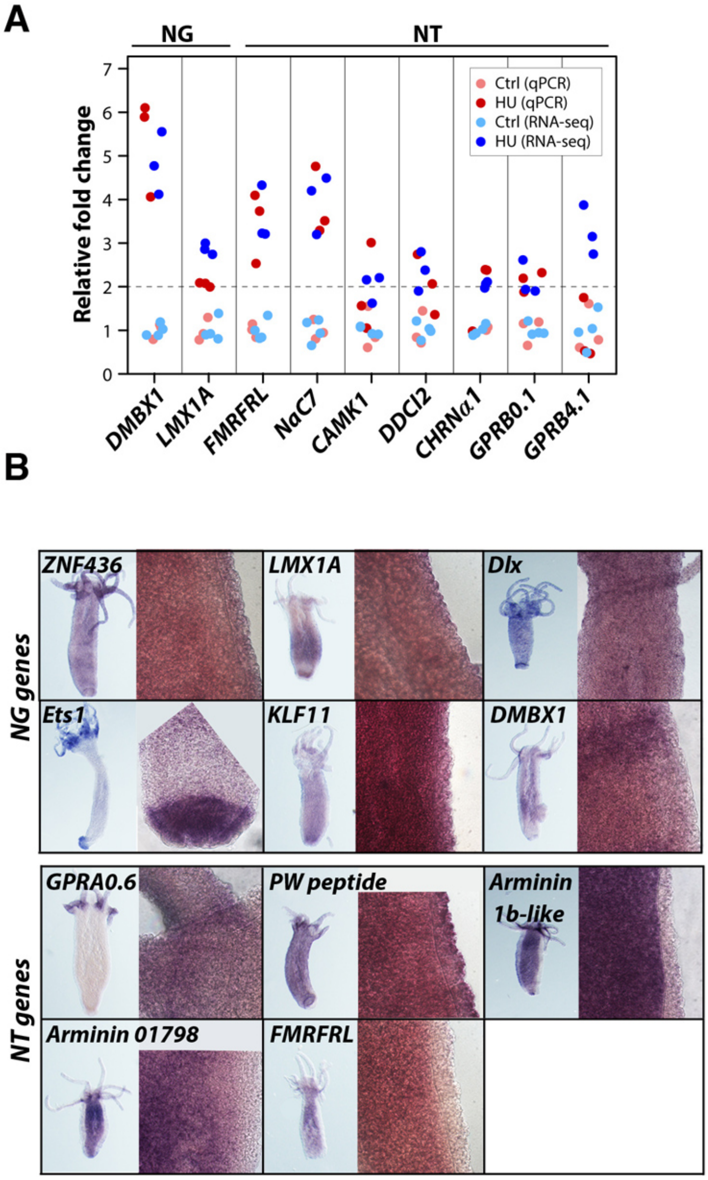

Supplt-10: (A) Q-PCR validation of the up-regulation of two NG and seven NT epithelial genes detected after the loss of neurogenesis. Real-time Q-PCR was performed on mRNA extracted seven days after HU withdrawal, corresponding to 10 days of starvation for control animals. The relative expression levels of the candidate genes were normalized against three housekeeping genes (*EF-1γ; TBP1, YWHAZ*). On this graph we represented the relative fold change in expression upon HU treatment assessed by both Q-PCR (pink-red dots) and RNA-seq (blue dots). The obtained data were multiplied by a factor as such as the average values of control conditions are equal to "1" for each gene. The horizontal dashed line represents the two-fold threshold. Note the good correlation between the RNA-seq and the qPCR quantifications.
(B) Confirmation of the epithelial expression of some neurogenic (*Dlx, DMBX1, Ets1, KLF11, LMX1A, ZNF436*) and neurotransmission (*Arminin 1b-1, Arminin 01798, FMRFRL,GPRA0.6, and PW peptide*) genes that are up-regulated after loss of neurogenesis. Some other genes did not provide a clearcut ISH pattern given their low level of expression in the body column (*Dlx1*, *CAMK1D, DDC-I2, NaC7, CHRNα1*).
