# Supplementary material for: Loss of neurogenesis in Hydra leads to compensatory regulation of neurogenic and neurotransmission genes in epithelial cells
Source: Philos Trans R Soc Lond B Biol Sci. 2016 Jan 5;371(1685):20150040. doi: 10.1098/rstb.2015.0040 (PMC4685579; doi:10.1098/rstb.2015.0040)
Supplement: Supplementary movies [file rstb20150040supp3.pptx]

## Slide 1
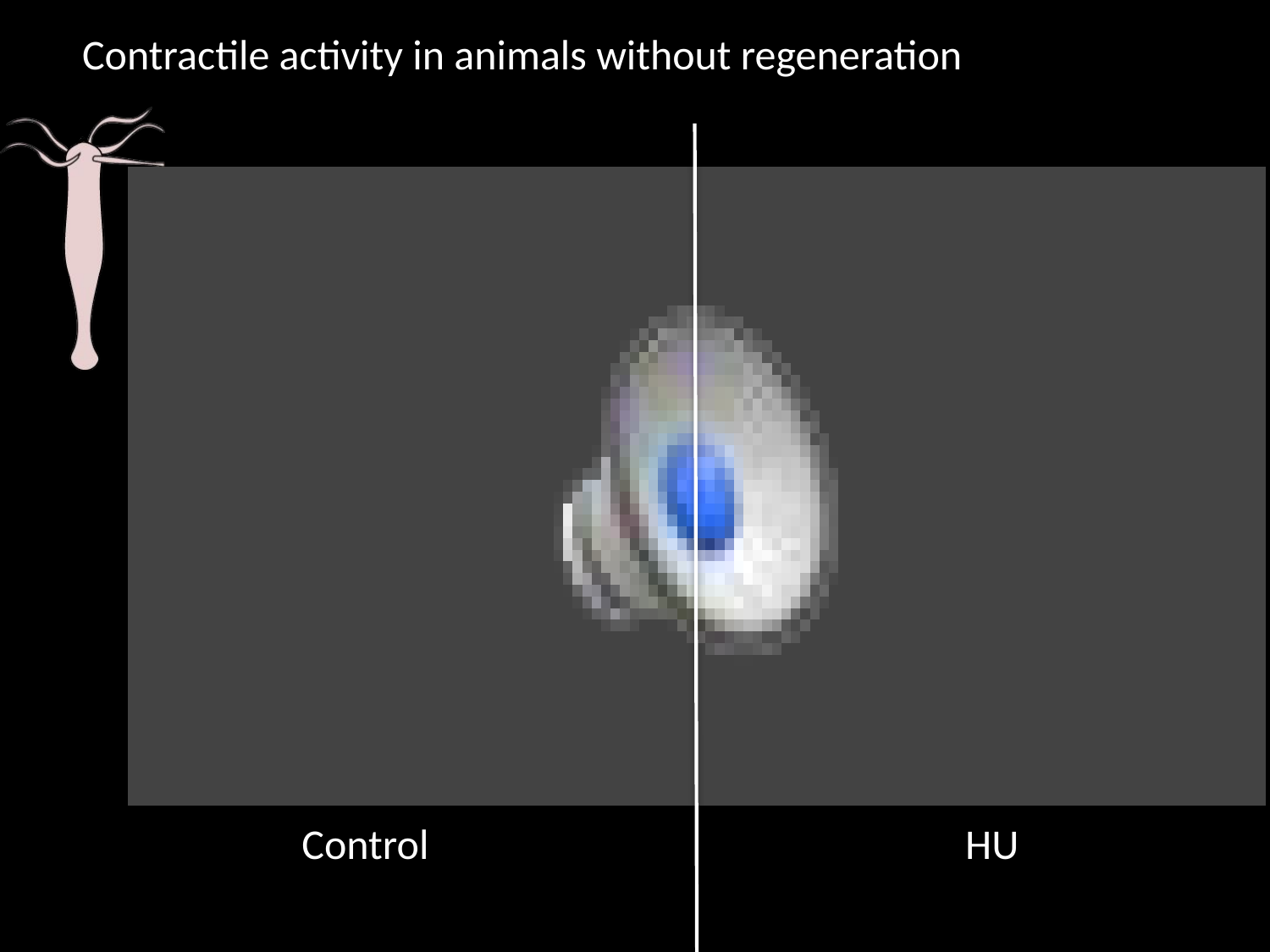

Contractile activity in animals without regeneration
Control
HU

## Slide 2
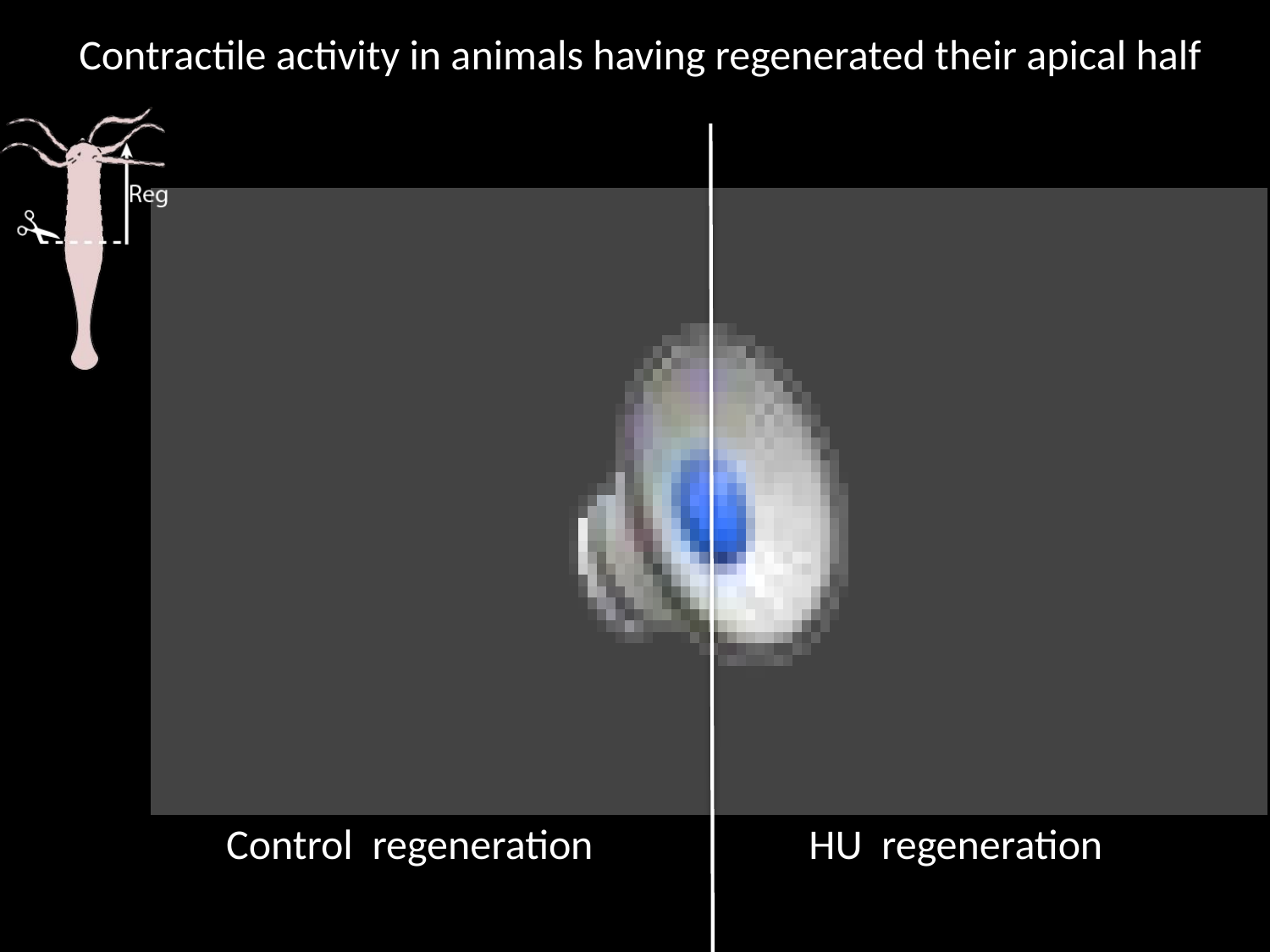

Contractile activity in animals having regenerated their apical half
Control regeneration
HU regeneration

## Slide 3
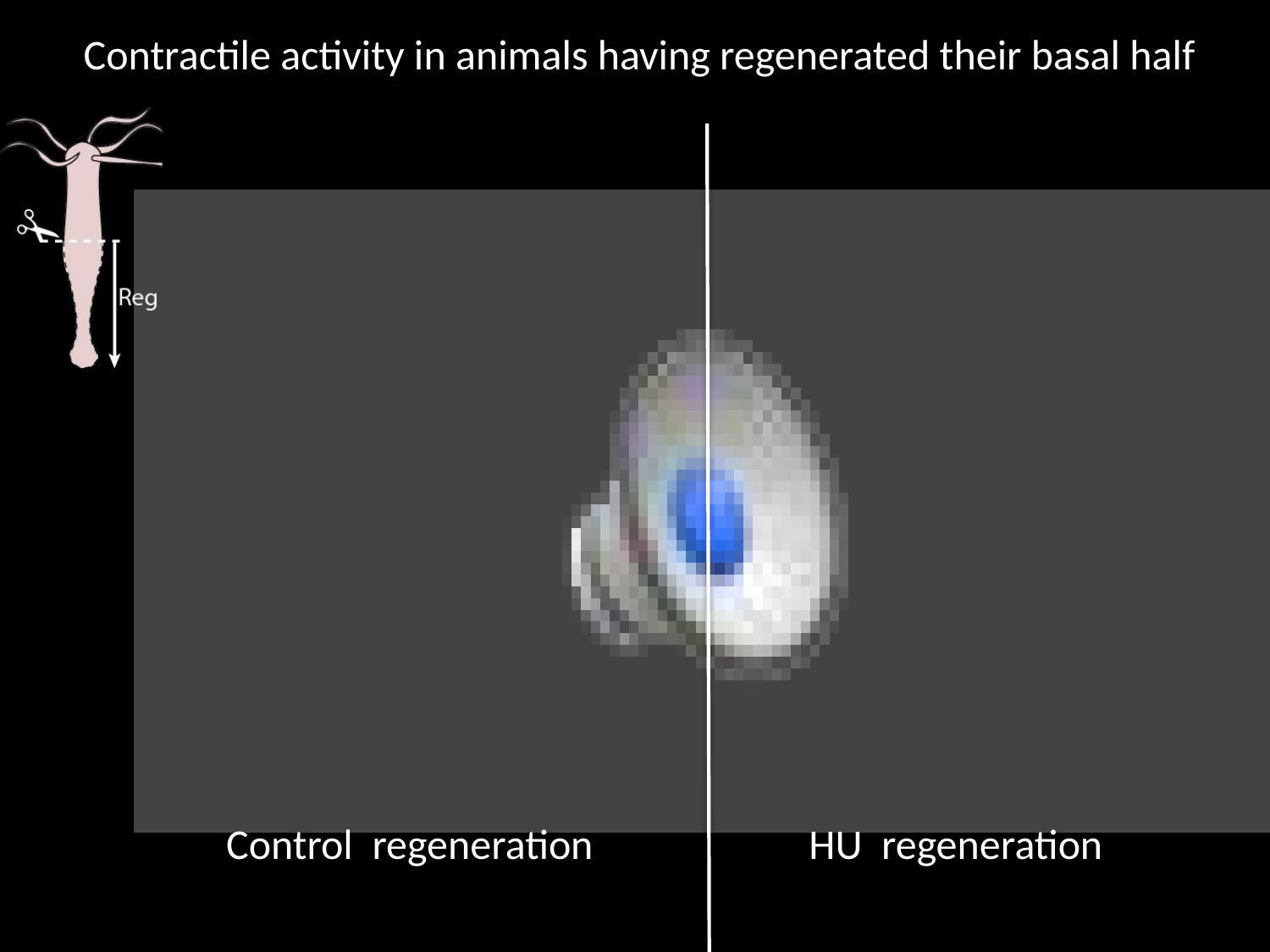

Contractile activity in animals having regenerated their basal half
Control regeneration
HU regeneration
